# Isoform-dependent lysosomal degradation and internalization of apolipoprotein E requires autophagy proteins

**DOI:** 10.1242/jcs.258687

**Published:** 2022-01-25

**Authors:** Gianna M. Fote, Nicolette R. Geller, Nikolaos E. Efstathiou, Nathan Hendricks, Demetrios G. Vavvas, Jack C. Reidling, Leslie M. Thompson, Joan S. Steffan

**Affiliations:** 1 UC Irvine Department of Biological Chemistry, 825 Health Sciences Road, Medical Sciences I, Room D240, UC Irvine School of Medicine, Irvine, CA 92697-1700, USA; 2 UC Irvine Department of Psychiatry and Human Behavior, Neuropsychiatric Center, UC Irvine Medical Center, 101 The City Drive South, Building 3, Route 88, Orange, CA 92868, USA; 3 Harvard Medical School Department of Ophthalmology, 243 Charles Street, Boston, MA 02114, USA; 4 Institute for Integrative Genome Biology, UC Riverside, Eucalyptus Drive, Riverside, CA 92521, USA; 5 UC Irvine MIND Institute, 2642 Biological Sciences III, Irvine, CA 92697-4545, USA; 6 UC Irvine Department of Neurobiology and Behavior, 2205 McGaugh Hall, Irvine, CA 92697, USA

**Keywords:** Alzheimer's disease, APOE, APOE4, Chaperone-mediated autophagy, LC3-associated endocytosis

## Abstract

The human apolipoprotein E4 isoform (APOE4) is the strongest genetic risk factor for late-onset Alzheimer's disease (AD), and lysosomal dysfunction has been implicated in AD pathogenesis. We found, by examining cells stably expressing each APOE isoform, that APOE4 increases lysosomal trafficking, accumulates in enlarged lysosomes and late endosomes, alters autophagic flux and the abundance of autophagy proteins and lipid droplets, and alters the proteomic contents of lysosomes following internalization. We investigated APOE-related lysosomal trafficking further in cell culture, and found that APOE from the post-Golgi compartment is degraded through autophagy. We found that this autophagic process requires the lysosomal membrane protein LAMP2 in immortalized neuron-like and hepatic cells, and in mouse brain tissue. Several macroautophagy-associated proteins were also required for autophagic degradation and internalization of APOE in hepatic cells. The dysregulated autophagic flux and lysosomal trafficking of APOE4 that we observed suggest a possible novel mechanism that might contribute to AD pathogenesis.

This article has an associated First Person interview with the first author of the paper.

## Abstract (precis)

**Summary:** Alzheimer's disease-associated isoform APOE4 accumulates in lysosomes and dysregulates autophagic flux. APOE can enter the lysosome through autophagy requiring LAMP2A, a receptor for chaperone-mediated autophagy.

## INTRODUCTION

Apolipoprotein E (APOE) facilitates lipid transport as a component of high density and very low density lipoproteins (Getz and Reardon, 2009), and is the major lipoprotein in the CNS (Fernandez et al., 2019). Humans have three major *APOE* alleles (*APOE2*, *APOE3* and *APOE4*) differing by only one or two amino acids (Huebbe and Rimbach, 2017). The *APOE4* allele is a powerful genetic risk factor for late-onset Alzheimer's disease (AD) (Crean et al., 2011), cardiovascular disease (Bennet et al., 2007) and reduced longevity (Abondio et al., 2019).

Many publications have investigated the effect of APOE4 on the degradation of amyloid β (Aβ), the main component of plaques found in the brains of AD patients (Castellano et al., 2011; Deane et al., 2008; Lin et al., 2018). However, few studies have focused on the degradation of APOE itself. APOE4 levels are lower than APOE3 in cultured cells (Lin et al., 2018), and in human serum, plasma and brain (Rasmussen et al., 2015). Reduced APOE plasma levels are associated with smaller hippocampal size in AD patients, especially in *APOE4* carriers (Teng et al., 2015), and it has been hypothesized that the use of APOE mimetics (Vitek et al., 2015) or increasing levels of APOE (Poirier et al., 2014) could be therapeutic for AD. Pulse-chase experiments show that APOE4 levels may be lower due to rapid degradation (Riddell et al., 2008), but the exact mechanism of this degradation is incompletely understood.

Although APOE is primarily secreted following processing in the Golgi, a portion of APOE is degraded by the lysosome instead. This lysosomal degradation has been observed in macrophages (Deng et al., 1995; Dory, 1991; Lucas and Mazzone, 1996), hepatocytes (Fungwe et al., 1999; Ye et al., 1992, 1993) and other cell lines (Ye et al., 1992, 1993), and occurs in the post-Golgi compartment, as evidenced by Brefeldin A (BFA) treatment, which collapses the Golgi and inhibits degradation of APOE (Deng et al., 1995; Ye et al., 1992). We sought to determine whether this post-Golgi lysosomal degradation requires autophagy proteins. Numerous autophagosomes (Nixon et al., 2005) and enlarged endosomes (Cataldo et al., 2000) accumulate in AD brain tissue, especially in *APOE4* carriers, suggesting that dysregulated trafficking of APOE through endosomes and/or autophagy may contribute to disease pathogenesis.

Autophagy is the process of trafficking cytosolic contents into the lysosome for degradation. There are three distinct mechanisms of autophagy, which function through partially overlapping sets of proteins; macroautophagy, microautophagy and chaperone-mediated autophagy (CMA) (Kaur and Debnath, 2015). Macroautophagy requires the formation of an autophagosome around cytosolic proteins and organelles; the autophagosome then fuses with the lysosome to release its contents. Microautophagy involves invagination of the endolysosomal membrane around target proteins. CMA involves direct internalization into the lysosome through a mechanism requiring lysosome-associated membrane protein 2A (LAMP2A, one of the three isoforms of LAMP2). A recent study has shown that CMA is inhibited in AD mouse models and downregulated in human AD brain tissue. In CMA-inhibited AD mouse models, APOE accumulated significantly in brain tissue. Neuronal knockout of either LAMP2A or ATG7 in wild-type mouse brain was sufficient to increase levels of APOE in the insoluble proteome, suggesting that multiple autophagic pathways might play a role in the degradation of APOE (Bourdenx et al., 2021). We sought to build on these *in vivo* observations by investigating *in vitro* whether intracellular lysosomal degradation of APOE requires autophagy proteins involved in autophagosome formation, autophagosome fusion or autophagosome-independent autophagy.

In addition to macroautophagy, microautophagy and CMA, several types of non-canonical autophagy have recently been defined. Non-canonical autophagy is the recruitment of lipidated LC3 family proteins (also known as the MAP1LC3 family) to endosomes (Heckmann et al., 2019) and phagosomes (Heckmann and Green, 2019), facilitating the lysosomal trafficking of proteins entering from the extracellular space. The autophagic protein–lipid conjugation machinery, autophagy-related proteins ATG5, ATG7, ATG12 and ATG16 (ATG16L1 and ATG16L2 in mammals), are required for non-canonical autophagy, together with Run domain Beclin-1 interacting and cysteine-rich domain containing protein (Rubicon), a protein that also suppresses canonical macroautophagy (Boyle and Randow, 2015; Martinez et al., 2015). Aβ was recently shown to enter cells through LC3-associated endocytosis (LANDO) (Heckmann et al., 2019). Since Aβ and APOE may compete for receptors to enter the cell through the same endocytosis pathways (Tachibana et al., 2019; Yamauchi et al., 2002), we investigated whether APOE can also enter the cell through non-canonical autophagy.

A thorough understanding of the autophagic trafficking of APOE could catalyze advances in the development of disease-modifying therapeutics for AD that target the abundance and activity of APOE4. Here, we found that in HEK293 cells stably expressing APOE proteins, that APOE4 expression stimulates macroautophagy but may ultimately reduce autophagic flux, and that this protein accumulates in enlarged lysosomes with altered proteomic contents compared to what is seen with APOE3, suggesting a possible contributing mechanism to loss of proteostasis observed in AD. We further analyzed the mechanism of lysosomal degradation of APOE in cellular models and found autophagy proteins LAMP2A, ATG7, syntaxin-17 (STX17) and Rubicon play a role in APOE degradation through canonical and non-canonical autophagy.

## RESULTS

### APOE4 expression alters autophagic flux and expression of autophagy proteins

APOE4 expression has previously been associated with lysosomal dysfunction (Persson et al., 2018; Prasad and Rao, 2018; Xian et al., 2018), and downregulation of autophagy (Parcon et al., 2017; Simonovitch et al., 2016). In order to investigate whether expression of APOE4–mCherry (mCh) can affect autophagic flux and expression of autophagy proteins, we developed HEK293 clonal cell lines stably expressing APOE3–mCh, APOE4–mCh or mCh only, which express similar amounts of APOE3 and APOE4 RNAs (Fig. S1Ai).

**Fig. 1.**
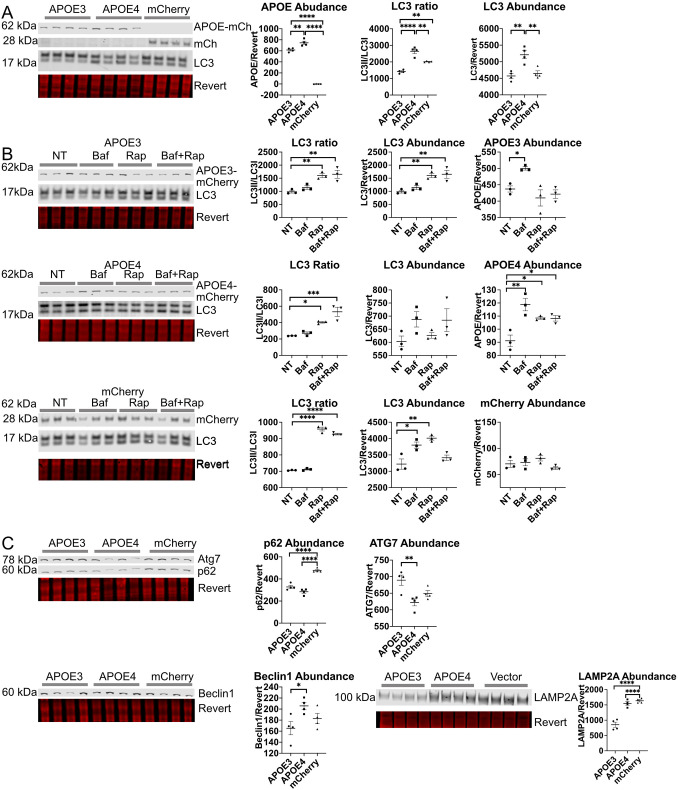
**APOE4 alters autophagic flux in in HEK293 cells stably expressing fluorescently tagged APOE.** (A,C) HEK293 cells expressing APOE3–mCh or APOE4–mCh or mCh vector were analyzed by western blotting. (B) HEK293 cells stably expressing APOE3–mCh, APOE4–mCh, or mCh vector were treated with Baf and analyzed as for A and C (50 nM 4 h), Rap (10 nM 4 h) or both. Quantitative results are mean±s.e.m. Revert, protein stain; NT, no treatment. *P<0.05, **P<0.01, ****P<0.0001 (one-way ANOVA with multiple comparisons correction).

The ratio of the amount of the lipidated form of LC3 proteins (LC3II) to the unlipidated form (LC3I) is often used as a marker of autophagic flux; cytosolic LC3I is conjugated to phosphatidylethanolamine to create LC3II when it associates with autophagosome membranes (Klionsky et al., 2016). APOE4–mCh cells had a significantly higher LC3II/LC3I ratio and significantly higher LC3 abundance (Fig. 1A). An increase in the LC3II/LC3I ratio may suggest either high macroautophagic flux, a late stage block of autophagy at the level of the lysosome causing autophagosome accumulation, or both. To discern between these possibilities, LC3 levels were assessed by western blotting following treatment with the commonly used autophagy inhibitor Bafilomycin A1 (Baf) (Klionsky et al., 2016), which de-acidifies the lysosome through inhibition of vacuolar-type ATPase (V-ATPase), and the mTOR inhibitor Rapamycin (Rap), which stimulates starvation-induced autophagy and is commonly used when assessing autophagic flux (Klionsky et al., 2016). Baf treatment significantly increased LC3 abundance in mCh-expressing cells, suggesting effective inhibition of the lysosome, but failed to significantly change LC3 abundance or ratio in APOE3–mCh- or APOE4–mCh-expressing cells (Fig. 1B), suggesting that there is already a late-stage autophagic block at the level of the lysosome. Rap increased LC3II/LC3I ratio in all three cell lines, suggesting effective stimulation of macroautophagy. However, LC3 abundance was increased with Rap only in APOE3–mCh and mCherry cells, with no change in APOE4–mCh cells. Compared directly to APOE3–mCh and mCh cells (Fig. 1B), APOE4–mCh cells have increased LC3 abundance at baseline (Fig. 1A), suggesting stimulated macroautophagy and a ceiling effect with Rap.

In order to further investigate dysregulation of autophagy in APOE4 cells, we assessed levels of autophagy proteins p62 (also known as SQSTM1), Beclin1, ATG7 and LAMP2A. Relative to APOE3–mCh cells, APOE4–mCh cells had increased Beclin1 protein, suggesting upregulation of macroautophagy, but ATG7 levels were significantly lower (Fig. 1C). p62 levels were reduced with expression of APOE3–mCh or APOE4–mCh compared to control, suggesting p62 is actively degraded by selective autophagy. Quantitative (q)PCR revealed no significant differences between the three cell lines in LC3B (MAP1LC3B), ATG7 or LAMP2A levels, and a paradoxical increase in Beclin1 transcript in APOE3 cells, suggesting that alterations in protein levels were not impacted by transcript levels (Fig. S1Ai–v). In summary, in APOE4–mCh cells, Rap treatment increases levels of Beclin1 but not LC3, suggesting macroautophagy upregulation, whereas Baf treatment has no effect on LC3 abundance or ratio, consistent with a late-stage macroautophagic block. A similar pattern of dysregulation has previously been observed as a compensatory mechanism in cells with impaired CMA in the context of expression of neurodegenerative disease-associated proteins (Xilouri et al., 2009). We evaluated levels of the CMA receptor protein LAMP2A, and found that LAMP2A levels were significantly increased in APOE4–mCh compared to APOE3–mCh cells, whereas there was no difference in LAMP2A between APOE4–mCh- and mCh-expressing cells (Fig. 1C). This may reflect high activity and turnover of LAMP2A in APOE3–mCh-expressing cells, whereas the altered endolysosomal pH observed previously with APOE4 expression (Xian et al., 2018) might reduce the turnover of LAMP2A, which depends on lysosomal acidification (Kaushik et al., 2008).

### APOE stably expressed in HEK293 cells is degraded by the lysosome in an isoform-specific manner

**Fig. 2.**
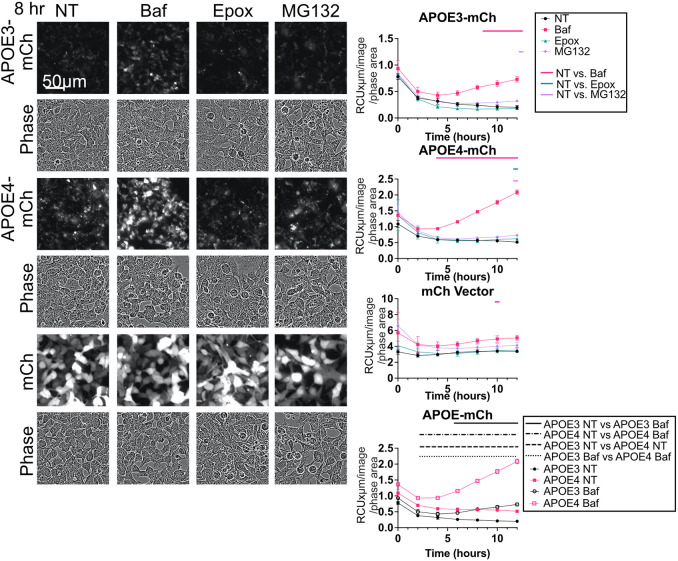
**APOE is turned over by autophagy in HEK293 cells with stable APOE expression.** Representative images and fluorescence intensity of APOE3–mCh, APOE4–mCh, or mCh cells treated with Baf (50 nM 4 h), Epox (100 nM), or MG132 (50 µM). Quantitative results are mean±s.e.m. Bars over graphs indicate time points at which *P*<0.05 on two-way ANOVA with post-hoc Dunnett test.

In order to determine whether APOE itself is degraded by the lysosome, APOE3–mCh and APOE4–mCh were assessed by western blotting and live-cell imaging following Baf or Rap treatment. Baf treatment significantly increased the abundance of APOE3–mCh and APOE4–mCh (Fig. 1B), and red fluorescence of APOE3–mCh and APOE4–mCh cells (Fig. 2). Baf did not change western blot levels or red fluorescence of mCh vector (Figs 1B and 2). Whereas Rap treatment significantly increased the LC3II/LC3I ratio (Fig. 1B), suggesting that LC3-dependent autophagy was effectively stimulated, Rap did not significantly reduce APOE levels. Instead, the abundance of APOE4–mCh significantly increased (Fig. 1B). APOE may therefore be turned over by a form of selective autophagy that functions independently of Rap, such as CMA (Finn et al., 2005; Mejlvang et al., 2018).

APOE4–mCh cells have significantly higher APOE levels by western blot (Fig. 1A) and fluorescence (Fig. 2) than APOE3–mCh, in spite of similar *APOE* RNA levels (Fig. S1Ai). There was a significant difference between slopes of APOE3–mCh and APOE4–mCh fluorescence treated with Baf over time when analyzed by linear regression, suggesting that APOE4–mCh fluorescence is more strongly affected by Baf and is turned over more rapidly by the lysosome. The proteasome inhibitors epoxomycin and MG132 did not significantly affect APOE-mCh levels at more than one time point (Fig. 2).

**Fig. 3.**
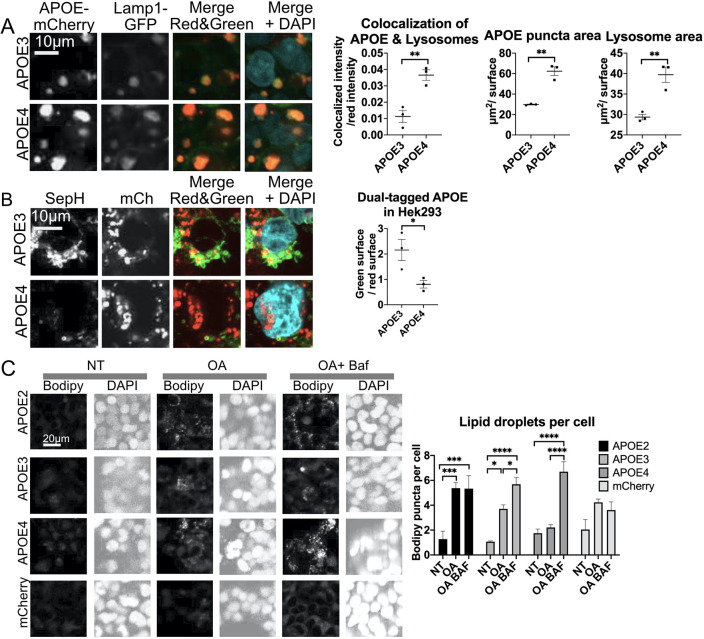
**APOE4 colocalizes with enlarged lysosomes.** (A) HEK293 cells stably expressing APOE3– or APOE4–mCh (red) and transfected with CellLight Lamp1–GFP (green), and stained with DAPI (blue). (B) HEK293 cells stably expressing APOE3– or APOE4–mCh–SepH. SepH shown in green. (C) HEK293 cells expressing APOE2, APOE3, APOE4 or vector were treated with oleic acid (OA, overnight) and Baf (4 h). NT, no treatment. Quantitative results are mean±s.e.m. In A–C, three wells were imaged per APOE isoform (three images per well) by confocal microscopy. Images were analyzed using Imaris software. Quantitative results are mean±s.e.m. *P<0.05, **P<0.01, ***P<0.001, ****P<0.0001 [Student's two-tailed unpaired *t*-test (A,B) or one-way ANOVA with a post-hoc Tukey–Kramer test (C)].

APOE3–mCh- and APOE4–mCh-expressing HEK cells were transiently transfected with CellLight Lamp1-GFP, a lysosomal marker, and imaged live using confocal microscopy. Imaris analysis revealed that APOE4–mCh cells had significantly higher colocalization between APOE and lysosomes, and significantly greater average surface area of individual lysosomal and APOE puncta (Fig. 3A) than did APOE3–mCh cells, suggesting greater trafficking to lysosomes. To further assess lysosomal localization, HEK293 cells stably expressing APOE3 or APOE4 dual-tagged with the fluorescent pH-sensitive tag SepHluorin (SepH) and mCh (APOE3–mCh–SepH and APOE4–mCh–SepH) were imaged live. This new APOE construct, generated in a published vector (Tanida et al., 2014), appears red in acidic lysosomes and green/yellow in the cytoplasm as expected (Koivusalo et al., 2010). APOE4–mCh–SepH cells had a significantly lower ratio of green to red fluorescence compared with APOE3–mCh–SepH cells, indicating a greater degree of lysosomal localization of the protein (Fig. 3B). These results suggest that although trafficking of APOE4–mCh to lysosomes appears to be intact, it may not be efficiently degraded, resulting in accumulation and higher abundance of APOE4–mCh than APOE3–mCh.

Another target of selective autophagy that may be affected by APOE isoform is cytoplasmic lipid droplets (LDs). Altered levels of LDs have previously been described in APOE4-expressing cells (Farmer et al., 2019; Liu et al., 2017), and in APOE4 compared to APOE2- or APOE3-expressing flies (Liu et al., 2017). We analyzed the number of LDs per cell in APOE2–, APOE3– and APOE4–mCh-expressing HEK293 cells to determine whether APOE4 expression affects LD formation or degradation. In order to stimulate LD formation, cells were treated with 200 μM oleic acid (OA) overnight, and cells were treated with Baf to block LD degradation by autophagy. Two-way ANOVA comparing APOE isoforms and treatment revealed an effect of treatment and an interaction between treatment and APOE isoform (Fig. 3C). An increase in LDs was observed in APOE2- and APOE3-expressing cells with OA, but no significant increase was observed in APOE4- or vector-expressing cells. However, when treated with Baf to block autophagy, LDs increased significantly in all APOE-expressing cells, including those expressing APOE4, but not in vector-expressing cells. This suggests that the number of LDs is low in APOE4 OA-treated cells not because formation of LDs is impaired, but because they are rapidly degraded, and when degradation is blocked with Baf, LD abundance is restored. Macroautophagy upregulation might cause depletion of LDs; however, the levels of LC3 appear to suggest that there may be a late-stage block in macroautophagy in these cells. LC3-independent microlipophagy has recently been described in mammalian cells (Schulze et al., 2020) and might be activated in APOE4-mCh-expressing cells.

### Autophagy of APOE requires ATG7, STX17 and LAMP2A in HepG2 cells

**Fig. 4.**
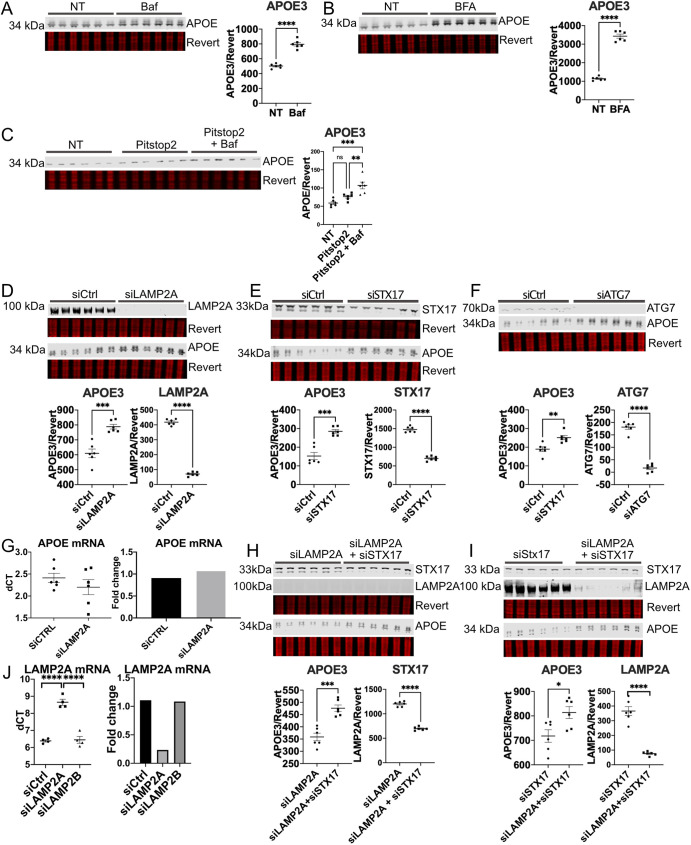
**APOE degradation requires autophagy proteins in HepG2 cells.** (A) HepG2 cells were treated with Baf (50 nM, 4 h) and analyzed by western blotting. (B) HepG2 cells were treated with BFA (5 μg/ml, 4 h). (C) HepG2 cells were treated with BFA and Baf and analyzed by western blotting. (D–F) HepG2 cell siRNA knockdown of (D) LAMP2A (E) STX17 or (F) ATG7 and analyzed by western blotting. siCtrl, control siRNA. (G) qPCR of HepG2 cells with siRNA against LAMP2A. (H,I) HepG2 cells with siRNA knockdown of LAMP2A, STX17, or both were analyzed by western blotting. (J) qPCR of LAMP2A or LAMP2B in HepG2 cells following LAMP2A siRNA. Revert, protein stain; NT, no treatment. *P<0.05, **P<0.01, ***P<0.001, ****P<0.0001 [Student's two-tailed unpaired *t*-test was used in western blot analysis, one-way ANOVA with post-hoc Tukey–Kramer test was used for qPCR].

APOE is strongly expressed in both liver and brain tissue *in vivo* (Xu et al., 2006; Srivastava and Bhasin, 1996), and immortalized human hepatic HepG2 cells express APOE3 endogenously. We used these cells to further investigate the mechanism of autophagic clearance of APOE3 with the lysosome de-acidifier Baf, which significantly increased APOE levels (Fig. 4A).

After synthesis in the endoplasmic reticulum (ER), APOE is trafficked to the Golgi to be modified by glycosylation and sialylation (Lee et al., 2010). BFA is a compound which disrupts Golgi trafficking, and causes collapse of the Golgi into the ER (Fujiwara et al., 1988). Several previous studies have found that BFA inhibits lysosomal trafficking of intracellular APOE in macrophages and hepatocytes (Deng et al., 1995; Ye et al., 1992). Consistent with these findings, we observed a significant increase in APOE3 levels in HepG2 cells with BFA treatment (Fig. 4B). To further visualize impaired lysosomal trafficking of APOE with BFA treatment, HEK293 cells stably expressing APOE3–mCh were transfected with the lysosomal marker GFP–Lamp1. APOE3–mCh forms puncta that colocalized with Lamp1–GFP, but with BFA treatment it loses its punctate pattern and is dispersed throughout the cell body (Fig. S1Bi). This dispersed pattern was also observed in HepG2 cells endogenously expressing APOE that were treated with BFA (Fig. S1Bii).

In order to determine whether some APOE enters the lysosome directly from the post-Golgi intracellular compartment, rather than being secreted and endocytosed, cells were treated with both Baf and Pitstop2, an inhibitor of clathrin-mediated endocytosis (von Kleist et al., 2011). In order to validate that Pitstop2 effectively inhibits endocytosis of APOE, HepG2 cells were treated with APOE3–mCh-containing medium from transfected HEK293T cells, which were selected based on their quick growth and consistently high yield of secreted APOE3-mCh following transfections (Fig. S1C). APOE3-mCh that enters HepG2 cells from conditioned medium is fluorescent red in the plane of greatest phase contrast (Fig. S1D). Pitstop2 significantly inhibited endocytosis of APOE3-mCherry from conditioned medium (Fig. S1D), whereas Latrunculin A, which inhibits phagocytosis, had no effect. Even when endocytosis was inhibited by Pitstop2, Baf significantly increased APOE levels (Fig. 4C), suggesting that APOE can reach the lysosome from the intracellular post-Golgi compartment instead of being secreted.

Having established that APOE is degraded by the lysosome in the post-Golgi compartment of HepG2 cells, we investigated whether APOE degradation requires autophagy proteins. siRNA against LAMP2A, ATG7 and STX17, were each separately transfected into HepG2 cells. APOE3 levels were significantly higher in HepG2 cells with knockdown of each of these autophagy proteins (Fig. 4D–F). LAMP2A knockdown did not affect APOE transcript level (Fig. 4G) or APOE secretion (Fig. S2A). To confirm that LAMP2A acts on APOE abundance by preventing localization of APOE to lysosomes, LAMP2A was knocked down in HEK293 cells expressing APOE3–mCherry (mCh) and the Lamp1–GFP lysosomal marker. Colocalization of APOE3–mCh with Lamp1–GFP was significantly reduced with LAMP2A knockdown (Fig. S2B). No significant change in overall green or red fluorescence was observed. Although LAMP2 regulates transcription of lipoprotein receptors and may participate directly in endocytosis (Leone et al., 2017; Qiao et al., 2020; Schneede et al., 2011), we found that LAMP2A knockdown had no effect on APOE3–mCh internalization from conditioned medium (Fig. S2C). Specificity of siRNA against LAMP2A but not LAMP2B was confirmed using qPCR, showing significant reduction of LAMP2A only (Fig. 4J).

Since LAMP2A is required for both CMA (Cuervo and Dice, 2000a) and for recruitment of STX17 to autophagosomes (Hubert et al., 2016), we performed a double-knockdown experiment with siRNA targeting both LAMP2A and STX17 in HepG2 cells. Knockdown of LAMP2A and STX17 together significantly increased APOE levels relative to knockdown of LAMP2A (Fig. 4H) or STX17 alone (Fig. 4I). This additive effect suggests that LAMP2A and STX17 may contribute to APOE degradation through two separate pathways, such as CMA and macroautophagy.

To further investigate whether macroautophagy plays a role in APOE degradation, we investigated whether APOE could be engulfed by LC3-positive vesicles. In HepG2 cells, 25% of LC3A- or LC3B-positive vesicles colocalized with endogenously expressed APOE. Baf treatment significantly increased colocalization and number of APOE spots per cell (Fig. S2D). We further investigated colocalization of transfected APOE–mCh and GFP–LC3A using HeLa cells, which were chosen due to their high adherence, transfection efficiency and flat morphology, which is ideal for imaging. A significantly greater degree of colocalization of APOE3–mCh and GFP–LC3A was observed (Fig. S2E) than for GFP–LC3A with mCh tag alone, and 3-dimensional visualization of *Z*-stack images revealed that APOE3–mCh was completely engulfed in some LC3-positive vesicles (Movie 2).

### APOE is also degraded by autophagy in immortalized neuronal cells

**Fig. 5.**
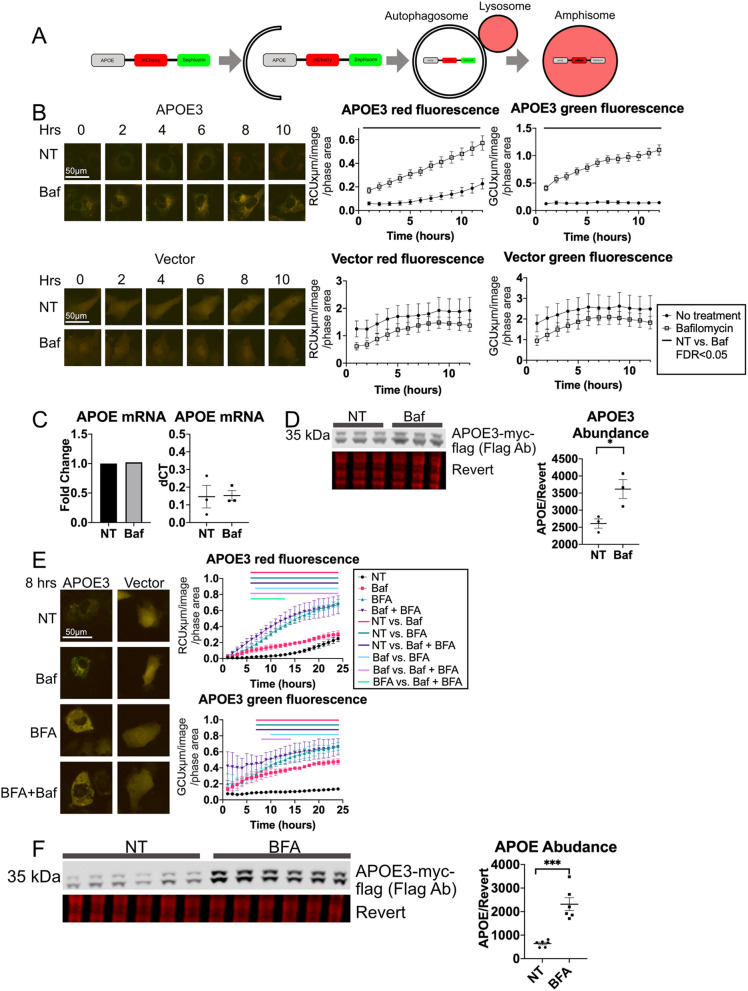
**APOE transiently overexpressed in ST14A cells is degraded by LAMP2A-dependent autophagy.** (A) Schematic of dual-tag fluorescent APOE with quenching of green SepHluorin in lysosomes. (B) APOE3–mCh–SepH and mCh–SepH tag fluorescence intensity in ST14A cells with Baf (50 nM, 4 h). (C) APOE3 mRNA in ST14A cells expressing APOE3–Myc–flag with Baf treatment. (D) APOE3–Myc–Flag abundance in ST14A cells following Baf treatment (4 h 50 nM). (E) APOE3–mCh–SepH fluorescence intensity following BFA (5 μg/ml) treatment. (F) ST14A cells expressing APOE3-myc-flag and treated with BFA (5 μg/ml, 4 h) were analyzed by western blotting. Quantitative results are mean±s.e.m. Revert, protein stain; NT, no treatment. *P<0.05, ***P<0.001 [Student's unpaired two-tailed *t*-test was used (D); bars above graphs indicate time points at which FDR<0.05 by two-way ANOVA with post-hoc Tukey–Kramer test (B,E)].

We next evaluated whether APOE is degraded through autophagic mechanisms in ST14A immortalized rat neuronally derived cells, similar to the mechanisms observed previously in macrophages and hepatocytes (Deng et al., 1995; Dory, 1991; Fungwe et al., 1999; Lucas and Mazzone, 1996; Ye et al., 1992, 1993), which we also observed in HepG2 cells. No endogenous APOE expression is detected in ST14A cells by western blot analysis, but transfected human APOE is detected (Fig. S2F). To confirm that transfected APOE is trafficked into lysosomes, we used the a pH-sensitive dual-tagged human APOE3–mCh–SepH construct as above (Fig. 5A). This fusion protein appears yellow in the cytoplasm due to colocalized fluorescence of both tags, and red at acidic pH, with quenching of the green pH-sensitive SepHluorin tag in the lysosome. Transfected ST14A cells initially appear yellow from fluorescence of both tag proteins, but over 12 h APOE3-expressing cells accumulate red puncta (Fig. 5B; Movie 1). The dual-tagged vector alone expresses brightly throughout the cytoplasm but cells do not accumulate red-only puncta (Fig. 5B).

Treatment with Baf prevents APOE3-expressing cells from forming red-only puncta, and red and green fluorescence levels increased significantly (Fig. 5B), indicating increased protein abundance. Baf had no effect on fluorescence in dual-tag vector-expressing cells (Fig. 5B). There were no changes in APOE RNA levels with Baf (Fig. 5C). To ensure that the results from live-cell imaging are not due to an artifact from the fluorescent tags, ST14A cells expressing APOE3 tagged only with Myc–Flag were treated with Baf for 4 h. Quantitative western blot analysis confirmed that APOE3 abundance was significantly increased by Baf treatment (Fig. 5D). We next examined the effect of Baf on ST14A cell internalization of APOE from conditioned medium. As in HepG2 cells, red APOE–mCh puncta failed to appear in Baf-treated cells (Fig. S3A). This rules out increased internalization in the presence of Baf as the cause of higher abundance of intracellular APOE. Baf treatment also had no effect on levels of APOE3–mCh–SepH in ST14A conditioned medium (Fig. S3D), suggesting that APOE secretion is not impaired, and accumulation upon Baf treatment is due to an inhibition of intracellular APOE lysosomal degradation.

To further investigate the internalized versus intracellular APOE that may accumulate with Baf, we examined post-translational modification of secreted and internalized APOE. After synthesis in the ER, APOE is trafficked to the Golgi where it can be modified by glycosylation and sialylation (Lee et al., 2010). APOE3–mCh–SepH recovered from whole HEK293T cell lysate (Fig. S3B) reveals a doublet, but only the upper band is secreted into the medium. When ST14A cells are treated with this conditioned medium, then lysed and analyzed by western blot, only the upper band is observed, suggesting that post-translational modification persists in the endolysosomal system following internalization (Fig. S3B). In order to determine whether this post-translational modification occurs only for transiently transfected tagged APOE, lysate and conditioned medium from human HepG2 hepatic cells, which express APOE3 endogenously, was analyzed by western blotting. An APOE doublet was also observed in HepG2 cells. Although both bands are observed in HepG2 cell lysate and in conditioned medium, the post-translationally modified band was more prominent in conditioned medium (Fig. S3C). We analyzed the unmodified lower band of APOE–Myc–Flag from the western blots presented in Fig. 5D, and found that there was a significant increase with Baf in this predominantly intracellular APOE species. This result indicates that a portion of APOE is trafficked to the lysosome before secretion and that APOE does not enter the lysosome solely through endocytosis.

### Golgi trafficking is required for autophagy of APOE in ST14A cells

To investigate whether Golgi trafficking is required for autophagy of APOE in ST14A cells, BFA was applied to APOE3–mCh–SepH-expressing ST14A cells. BFA eliminated all red puncta, and cells remained homogenously yellow, although BFA has no known effect on lysosomal acidification (Fig. 5E). When BFA and Baf are applied in combination, cells appear identical to those subjected to BFA-only treatment, without the yellow punctate pattern observed in Baf cells, suggesting that BFA blocks lysosomal trafficking upstream of the de-acidifying effects of Baf. APOE3 red and green fluorescence and abundance by western blot increased with BFA (Fig. 5E,F). These results suggest that, as previously demonstrated in hepatic cells, a portion of APOE expressed in neuron-like cells is also degraded by the lysosome in the post-Golgi, pre-secretion intracellular compartment.

### LAMP2A also facilitates APOE degradation in neuron-like ST14A cells and *in vivo*

**Fig. 6.**
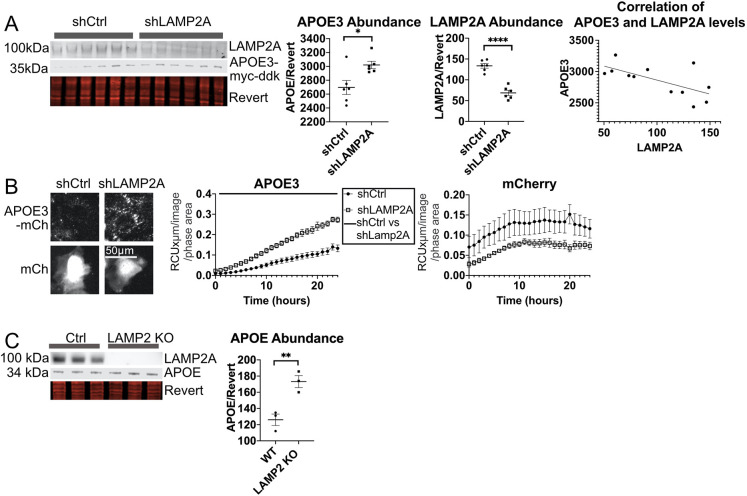
**LAMP2A is required for autophagy of APOE3 in ST14A cells.** (A,B) ST14A cells were co-transfected with shRNA targeting LAMP2A and (A) APOE3–Myc–Flag or (B) APOE3–mCh, and APOE3 levels were assessed by western blot or fluorescence intensity. shCtrl, control shRNA. Bars above graph in B indicates time points at which FDR<0.05 by two-way ANOVA. (C) APOE levels in mouse brain tissue from 2-year-old wild-type and LAMP2 knockout mice. Quantitative results are mean±s.e.m. *P<0.05, **P<0.01, ****P<0.0001 [Student's two-tailed unpaired *t*-test (A,C)].

In order to determine whether autophagy proteins ATG7 and LAMP2A are required for degradation of APOE in neuron-like cells, as in HepG2 cells, shRNA against ATG7 or LAMP2A was transfected into ST14A cells co-transfected with tagged APOE3–Myc–Flag. APOE3–Myc–Flag protein levels were significantly increased by LAMP2A knockdown (Fig. 6A), and levels of APOE were negatively correlated with LAMP2A levels (Pearson's correlation coefficient −0.65, *P*<0.02). The fluorescence intensity of APOE3–mCh increased significantly with LAMP2A knockdown (Fig. 6B). Surprisingly, ATG7 knockdown (Fig. S4A) did not show a significant increase of APOE3 levels in ST14A cells, suggesting that the major autophagic mechanism in ST14A cells may not be LC3-dependent, consistent with this being CMA.

We next examined potential alternative mechanisms, besides changes in lysosomal degradation, for the observed increase in APOE levels following LAMP2A knockdown by evaluating APOE expression, secretion and internalization. qPCR revealed no change in APOE mRNA levels with LAMP2A knockdown in ST14A cells (Fig. S5A). APOE protein levels in medium from ST14A cells with LAMP2A knockdown were significantly higher than from cells without LAMP2A knockdown, consistent with impaired degradation (Fig. S5B). LAMP2A knockdown in our system had no effect on APOE3–mCh internalization from APOE3–mCh conditioned medium (Fig. S5C).

To evaluate whether LAMP2 is also required for APOE degradation in brain tissue *in vivo*, whole brain tissue from LAMP2 knockout and wild-type mice aged 2 years (Tanaka et al., 2000) was harvested, lysed, and analyzed by western blotting. APOE protein levels were significantly increased in LAMP2-knockout brain (Fig. 6C), consistent with reduced APOE degradation. Although we cannot rule out the possibility that loss of LAMP2B may contribute to APOE accumulation in these mice, these results are in agreement with a recent study that found that knockout of LAMP2A in neurons *in vivo* is sufficient to increase insoluble APOE protein levels (Bourdenx et al., 2021).

### Internalization of fluorescently tagged APOE is isoform dependent

Previous work suggests that endocytosis is dysregulated by APOE4; APOE4 impairs endosomal receptor recycling and APOE recycling (Chen et al., 2010; Heeren et al., 2004; Xian et al., 2018), causes transcriptional dysregulation of endosomal pathways (Nuriel et al., 2017), and alters endosomal morphology so they are either larger or smaller depending on tissue or cell type (Cataldo et al., 2000; Lin et al., 2018; Narayan et al., 2020). We hypothesized that the defects in the endocytic pathway observed with APOE4 may be the result of altered internalization of APOE4 itself. In order to study isoform-specific internalization of APOE, conditioned medium was collected from HEK293T cells transfected with APOE2–mCh, APOE3–mCh or APOE4–mCh. APOE2–mCh was included in this experiment as a negative control due to the known lower affinity of APOE2 for LDL receptors resulting in reduced endocytosis (Yamamoto et al., 2008). No significant difference in APOE levels in the media was detected between isoforms in each conditioned medium (Fig. S6A). Native gel electrophoresis revealed that fluorescently tagged APOE secreted from HEK293T cells into medium (Fig. S6B) displays a pattern distinct from un-lipidated APOE and similar to published examples of lipidated APOE (Hubin et al., 2019). No difference in size or pattern was observed between APOE isoforms, suggesting that in this model APOE4 does not have altered aggregation or lipidation relative to the other isoforms. Similar results have been found previously in a similar system of transfected HEK cells expressing and secreting APOE2, APOE3 and APOE4 (Huang et al., 2019).

**Fig. 7.**
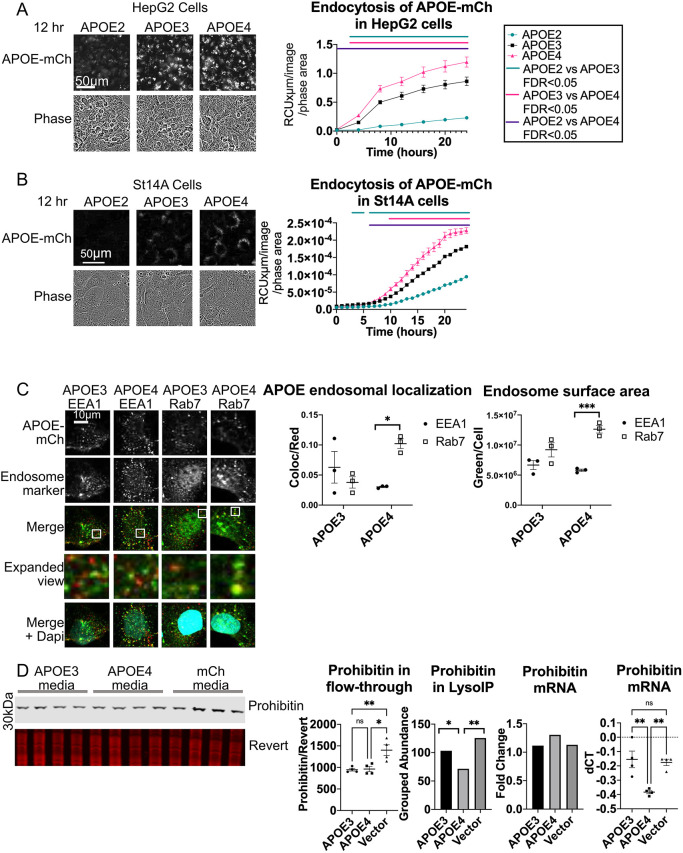
**Fluorescently tagged APOE is endocytosed in an isoform-dependent manner and alters endosomal morphology.** (A,B) APOE–mCh-conditioned medium was collected from HEK293T cells and applied to (A) HepG2 and (B) ST14A cells. Cells were imaged and red fluorescence/phase area calculated. Bars represent times when FDR<0.05 between APOE isoforms by two-way ANOVA. (C) ST14A cells were treated for 24 h with conditioned medium with APOE3–mCh or APOE4–mCh (red), and immunocytochemistry for EEA1 or Rab7 (green) was performed, and cells stained with DAPI (blue). Cells were imaged by confocal microscopy and analyzed using Imaris image. Magnified view indicates enlarged images from white boxes in merge panel. (D) HepG2 cells were treated for 24 h with APOE3–mCh or APOE4–mCh conditioned medium, lysosomes were immunoprecipitated and proteomic contents analyzed by mass spectrometry. Proteins reduced in APOE4 lysosomes that were not reduced in mCh-treated cells included mitochondrial proteins such as prohibitin. Western blot analysis for prohibitin was performed on lyso-depleted flow-through. Revert, protein stain. HepG2 cells treated with APOE-mCh conditioned medium were also analyzed by qPCR. Quantitative results are mean±s.e.m. *P<0.05, **P<0.01, ***P<0.001; ns, not significant (one-way ANOVA with Tukey–Kramer test).

APOE2, APOE3 or APOE4–mCh conditioned medium was next applied to HepG2 liver cells (Fig. 7A), or ST14A cells (Fig. 7B). In both cell types, intracellular red fluorescence appeared in a granular pattern and increased over time, consistent with internalization and continuing fluorescence due to the pH-resistant nature of the mCh tag. Internalization levels were isoform-dependent, with the highest fluorescence observed in both cell types treated with APOE4–mCh, and the lowest in APOE2–mCh-treated cells (Fig. 7A,B).

### APOE4 associates with enlarged late endosomes and alters the proteomic contents of lysosomes following internalization

To determine whether internalization of fluorescent APOE is sufficient to induce a change in endosome morphology in ST14A cells, APOE3–mCh or APOE4–mCh conditioned medium was applied to ST14A cells for 24 h. Cells were fixed and stained with antibodies against mCh and early endocytic marker EEA1 or the late endocytic marker Rab7 (Fig. 7C). APOE4 colocalized with late endosomes significantly more than with early endosomes, whereas APOE3 did not. There was also a significantly higher intensity of Rab7 staining per cell in APOE4-treated cells (Fig. 7C), suggesting that late endosomes may be enlarged by internalization of APOE4.

We further investigated the impact of APOE4 on the endolysosomal system by analyzing the proteomic contents of lysosomes from HepG2 cells following internalization of APOE3–mCh, APOE4–mCh, or mCh conditioned medium. A HepG2 cell line stably expressing HA-tagged TMEM192, a lysosomal membrane protein that can be used for immunoprecipitation of intact lysosomes (Fig. S7A) (Abu-Remaileh et al., 2017), was generated for this experiment. Proteomic contents of lysosomes were analyzed using mass spectrometry and compared statistically by one-way ANOVA (Table S1). Enrichment of lysosomes in the LysoIP fraction was verified by western blot (Fig. S7B). We identified proteins that were uniquely altered in APOE4-treated cells relative to APOE3-treated cells, and were not significantly altered between mCh- and APOE3-treated cells in the same direction (Fig. S7C). Over half of the proteins reduced in APOE4 lysosomes that were not reduced in mCh treated cells were mitochondrial proteins, including prohibitin, a component of a complex that functions as a mitophagy receptor (Wei et al., 2017) (Fig. 7D; Fig. S7D, Table S1), suggesting that mitophagy may be impaired by the presence of APOE4 in the endolysosomal system. Western blot analysis of the lysosome-depleted flow-through showed no significant difference in prohibitin protein levels between APOE3 and APOE4-treated cells, and prohibitin mRNA was significantly increased in APOE4 cells relative to APOE3- or mCh-treated cells (Fig. 7D). This suggests that the depletion of prohibitin from the LysoIP eluate in APOE4 relative to APOE3-treated cells is specific to the lysosome-enriched fraction, and does not reflect overall reduced abundance. Proteins enriched in the lysosome of APOE4-containing cells included a variety of secretory and endocytosis pathway proteins, including several resident Golgi proteins, membrane-tethering proteins and extracellular matrix proteins (Fig. 7D; Fig. S7D, Table S1). Thus, APOE4 appears to inhibit the lysosomal trafficking of mitochondria while enhancing the lysosomal degradation of secretory or endocytic vesicles.

### Internalization of APOE requires ATG7 and Rubicon

**Fig. 8.**
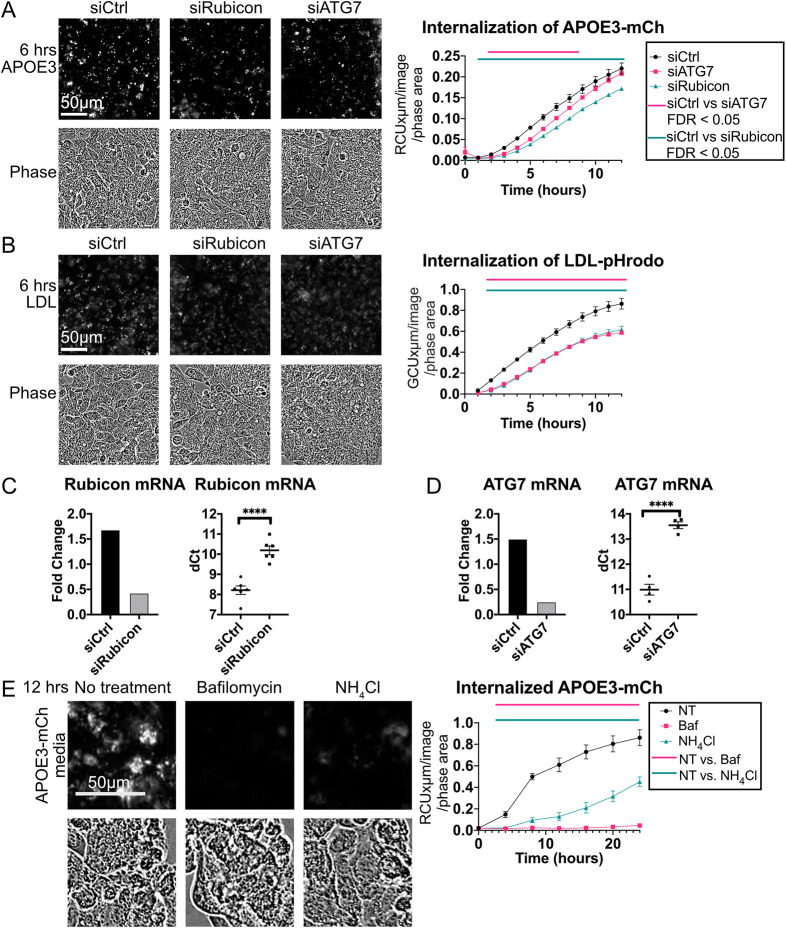
**Knockdown of Rubicon or ATG7 reduces APOE internalization.** (A,B) ATG7 and Rubicon were knocked down using siRNA in HepG2 cells, and (A) conditioned medium with APOE3–mCh or (B) medium containing LDL-pHrodo was applied. siCtrl, control siRNA. (C,D) HepG2 cells with siRNA knockdown of (C) ATG7 or (D) Rubicon were analyzed by qPCR. (E) HepG2 cells were treated with inhibitors and APOE3–mCh conditioned medium. Concentrations of inhibitors used were: 50 nM Bafilomycin A1, 20 mM ammonium chloride. NT, no treatment. Cells were imaged and fluorescence quantified by incucyte. Imaging and fluorescence quantification by incucyte. Quantitative results are mean±s.e.m. ****P<0.0001 [Student's two-tailed unpaired *t*-test (C,D); bars represent times when FDR<0.05 between APOE isoforms by two-way ANOVA with a post-hoc Dunnett's test (A,B,E)]

We hypothesized that LC3-associated endocytosis may contribute to APOE internalization. In addition to the ATG8 conjugation machinery (ATG3, ATG5, ATG7, ATG12 and ATG16L), LC3-associated endocytosis requires Rubicon. Rubicon can divert the Class III PI3K complex (PI3KC3) away from its function of initiating autophagosome formation, activating non-canonical autophagy and inhibiting macroautophagy instead (Wong et al., 2018). To determine whether LANDO contributes to APOE internalization, Rubicon and ATG7 were targeted with siRNA in HepG2 cells. Both Rubicon and ATG7 knockdown significantly reduced internalization of APOE3 (Fig. 8A), and similarly reduced internalization of LDL-phrodo (Fig. 8B). Knockdown of ATG7 and Rubicon were verified by qPCR (Fig. 8C,D) and western blotting (Fig. 4F; Fig. S8A).

Although inhibition of LANDO does not inhibit the internalization of Aβ initially, endocytosis over time is reduced due to impaired receptor recycling (Heckmann et al., 2019; Lucin et al., 2013). Several studies have shown that acidification of endosomes and lysosomes by functional v-ATPase is required for the recruitment of LC3 in non-canonical autophagy processes (De Faveri et al., 2020; Florey et al., 2015; Gao et al., 2016), and the v-ATPase inhibitor Baf impairs recycling of endocytic receptors (Johnson et al., 1993; Presley et al., 1997). Treatment of HepG2 cells with lysosomal de-acidifiers Baf or ammonium chloride (NH_4_Cl) significantly reduces internalization of APOE3–mCh (Fig. 8E), suggesting that reduced recycling of receptors may underlie the reduced APOE3–mCh fluorescence observed. A possible alternative mechanism leading to reduced fluorescence is that autophagy is stimulated by Rubicon knockdown to degrade more APOE3–mCh. In order to rule this out, a double knockdown of both Rubicon and ATG7 was performed (Fig. S8A). If Rubicon reduced fluorescence by stimulating macroautophagy, ATG7 knockdown would be expected to reduce the effect of Rubicon knockdown. To the contrary, we observed the same reduction of APOE3–mCh fluorescence in Rubicon and Rubicon plus ATG7 knockdown cells, suggesting that these two knockdowns reduce fluorescence through the same mechanism (Fig. S8B). In order to directly visualize recruitment of LC3 family proteins to APOE-containing endocytic compartments in a LANDO-like process, ST14A cells, chosen for their flat morphology, which is ideal for imaging, were treated with conditioned medium containing APOE3–mCh and APOE4–mCh. Colocalization of APOE with LC3A and/or LC3B was observed, with 17% of LC3A and LC3B spots colocalizing with APOE3–mCh and 22% colocalizing with APOE4–mCH spots (Fig. S8C). Taken together, these results suggest that in addition to being degraded by LAMP2A-dependent autophagy, APOE can be internalized in an LC3-associated process.

## DISCUSSION

In this study, we found that APOE4 has increased lysosomal trafficking and accumulates in enlarged lysosomes, reflecting impaired clearance despite activated macroautophagy. APOE4 alters abundance of autophagy proteins and the proteomic contents of lysosomes following internalization, suggesting a possible contributing mechanism to the loss of proteostasis observed in AD. We further demonstrated that APOE is degraded by autophagy requiring LAMP2A in HepG2 and ST14A cells, and LAMP2 *in vivo* in mouse brain tissue, supporting a role for CMA in the degradation of APOE. In HepG2 cells, macroautophagy-associated proteins were also required for autophagic degradation and efficient internalization of APOE, suggesting that several autophagic pathways contribute to autophagy of APOE.

### Lysosomal trafficking of APOE4 is increased, dysregulating autophagy

Expression of APOE4 results in enlarged endosomes in mouse models (Nuriel et al., 2017), patient brains (Cataldo et al., 2000) and induced pluripotent stem cell (iPSC)-derived neuronal cells (Lin et al., 2018). APOE4 causes dysregulated endo/lysosomal pH (Prasad and Rao, 2018; Xian et al., 2018) and damages lysosomal membrane integrity (Persson et al., 2018). Consistent with these studies, we found that, although fluorescent imaging shows increased localization of APOE4 to lysosomes and late endosomes, APOE4 accumulates and these acidic compartments swell, suggesting that lysosomal degradation is impaired. We have found that APOE3 is cleared by LAMP2A-dependent autophagy, suggesting that CMA might play a role in APOE3 clearance. It is possible that APOE4 is not efficiently degraded by CMA, resulting in higher APOE4–mCh levels with compensatory activation of macroautophagy as evidenced by reduced p62, higher Beclin1 and a high LC3II/LC3I ratio. Similarly, α-synuclein protein is normally degraded by CMA, but the mutant Parkinson's disease-associated form blocks CMA, causing compensatory upregulation of macroautophagy resulting in accumulation of autophagosomes, lysosomal membrane destabilization, and autophagic cell death in neurons (Wu et al., 2014; Xilouri et al., 2009). A similar situation may occur in AD patients with APOE4 expression, resulting in neurodegeneration.

Recent studies have shown that APOE4 downregulates autophagy; APOE4-expressing mouse-derived astrocytes have reduced autophagic flux (Simonovitch et al., 2016), and APOE4 directly inhibits expression of autophagy-related genes by binding DNA CLEAR motifs (Parcon et al., 2017). We observed higher levels of LC3II in APOE4-expressing cells (Fig. 1A), which can be interpreted as either activated autophagy with increased LC3 lipidation, or impaired autophagy with a block in autophagosome degradation. We observed in Fig. 2A–C that Baf does not significantly affect LC3 levels in APOE-expressing cells, and Rapamycin significantly increases the LC3II/LC3I ratio, suggesting that there might be a late-stage block in autophagic flux at the level of the lysosome. This block may be exacerbated by activation of macroautophagy at the initiation stage, as evidenced by increased Beclin1 levels and increased LC3 abundance, similar to the effect of Rapamycin. Degradation of a specific autophagic substrate, LDs, seems to be enhanced in these APOE4-expressing cells. Activated macroautophagy might require increased lipophagy to supply the necessary lipids to form autophagosomes (Ogasawara et al., 2020), and autophagic degradation of LDs may proceed even when autophagosome fusion is impaired through LC3-independent microlipophagy (Schulze et al., 2020).

In addition to examining autophagic flux in cells expressing APOE4, we found that internalization of APOE4 might be sufficient to dysregulate some autophagic processes. Mitochondrial proteins have reduced abundance in lysosomes of cells endocytosing APOE4, consistent with the reduced mitophagy shown in APOE4-expressing mice and primary astrocytes (Schmukler et al., 2020; Simonovitch et al., 2019). We also identified several proteins that were increased in the lysosomes of cells endocytosing APOE4, including syntaxin-6 (STX6), a soluble N-ethylmaleimide-sensitive factor attachment protein receptor (SNARE) protein. This protein is critical for fusion of endosomes and certain types of autophagic vesicles (Nozawa et al., 2017) and an increased level in the lysosome may reflect upregulated autophagic activity. STX6 was recently identified by proteomics as being dysregulated in AD brain tissue, with a potentially causal role in pathology (Wingo et al., 2021). Our LysoIP experiments were performed in HepG2 cells which endogenously express APOE3, and thus are a model of heterozygous APOE4 expression, where both APOE4 and APOE3 co-exist in the endolysosomal system.

### APOE can be degraded by the lysosome in the post-Golgi compartment

The lysosomal degradation of APOE in the post-Golgi compartment has previously been observed in macrophages (Deng et al., 1995; Dory, 1991; Lucas and Mazzone, 1996) and hepatocytes (Fungwe et al., 1999; Ye et al., 1992, 1993). We used a novel pH-sensitive fluorescent APOE3 construct to demonstrate that APOE can also be degraded by the lysosome in the post-Golgi compartment of neuron-like cells. Previous studies investigating lysosomal degradation of APOE that occurs intracellularly instead of secretion (Deng et al., 1995; Dory, 1991; Lucas and Mazzone, 1996) utilized BFA, a compound that blocks COPI coat association with the Golgi, ultimately causing collapse of the Golgi (Fujiwara et al., 1988). These studies concluded that because BFA blocked APOE degradation, APOE is trafficked to the lysosome from the post-Golgi compartment. Similarly, insensitivity to BFA has been used to conclude that autophagic degradation of procollagen chains occurs at the ER exit site, before entry into the Golgi (Omari et al., 2018). However, reports conflict on whether BFA impairs trafficking from endosomes to lysosomes (Lippincott-Schwartz et al., 1991; Wood and Brown, 1992), and one report shows that BFA also impairs the autophagy of endoplasmic reticulum (Wang et al., 2018). Thus, while our results suggest that autophagy of APOE occurs in the post-Golgi compartment, we cannot completely exclude the possibility that BFA impairs autophagy of APOE at the ER or by affecting the lysosome itself.

The autophagic mechanism of Golgi degradation has not been thoroughly characterized, although transport from Golgi into lysosomes has been observed (Ashok and Hegde, 2009; Black and Pelham, 2000; Briant et al., 2017; Chu et al., 2014; Gelling et al., 2012; Hellerschmied et al., 2019; Tewari et al., 2014, 2015; Wolins et al., 1997) and Golgi components found in autophagosomes (Lu et al., 2020). All of the ER-to-lysosome-associated degradation pathways that have been defined thus far require LC3 (Chino and Mizushima, 2020; Fregno et al., 2018; Fregno and Molinari, 2019; Ishida et al., 2009; Omari et al., 2018), whereas autophagy of APOE in ST14A cells is not affected by knockdown of ATG7. However, several types of autophagy do not require LC3, including CMA, lysosomal degradation of mitochondria-derived vesicles (Soubannier et al., 2012) and microlipophagy (Schulze et al., 2020; Vevea et al., 2015). Future studies will be required to confirm whether autophagic degradation of APOE occurs at the Golgi, as our results suggest, or at the ER through a novel LC3-independent form of ER-associated lysosomal degradation.

### APOE degradation requires LAMP2A, and ATG7-dependent autophagy and LANDO of APOE occur in HepG2 cells

APOE is a secreted protein that is trafficked through the Golgi and then released into the extracellular space (Takacs et al., 2017) or degraded by the lysosome (Deng et al., 1995; Dory, 1991; Fungwe et al., 1999; Lucas and Mazzone, 1996; Ye et al., 1992, 1993). We found that LAMP2A was required for this lysosomal degradation of APOE in cells, consistent with its degradation by CMA. An increase in APOE secretion was observed with LAMP2A knockdown in ST14A but not HepG2 medium, possibly because of robust transient overexpression of APOE in ST14A cells, which may be more prone to secrete excess APOE than sequester it intracellularly.

A recent study showed that LAMP2A-dependent CMA is inhibited in AD mouse models and downregulated in human AD brain tissue. In LAMP2A-KO AD mouse models, APOE accumulated significantly in brain tissue (Bourdenx et al., 2021). Several single nucleotide polymorphisms (SNPs) in the *LAMP2* gene are associated with significantly increased AD risk in human male APOE4 carriers, further suggesting that LAMP2-dependent autophagy of APOE4 may contribute to pathology (Kristen et al., 2021).

LAMP2 has three isoforms, LAMP2A, LAMP2B and LAMP2C, distinguished by variation in their short C-terminal cytosolic tail. Both LAMP2A (Hubert et al., 2016) and LAMP2B (Chi et al., 2019) are implicated in autophagosome–lysosome fusion, the former in a STX17-dependent manner. However, LAMP2A also functions in CMA without an autophagosome (Cuervo and Dice, 2000a). When we performed dual-knockdown of LAMP2A and STX17, we observed an additive effect on APOE accumulation, suggesting that these autophagy proteins act at least in part through independent autophagic mechanisms. If the LAMP2A-dependent mechanism that degrades APOE is CMA, it would be surprising that a secreted protein such as APOE could escape the secretory system into the cytosol to be recognized by Hsc70. There is some literature showing that APOE can escape into the cytoplasm in neurons (Chang et al., 2005; Huang et al., 2001; Lovestone et al., 1996) and hepatocytes (Hamilton et al., 1990), although one report did not observe cytoplasmic escape of APOE (DeMattos et al., 1999). APOE has also been observed outside the secretory system in mitochondria (Nakamura et al., 2009), mitochondria-derived vesicles (Roberts et al., 2021) and the nucleus (Parcon et al., 2017; Theendakara et al., 2016).

We found that ST14A cells also require LAMP2A, but not ATG7 to degrade APOE. APOE degradation mechanisms may be cell type dependent, occurring in neuron-like cells primarily through CMA or microautophagy, and in other cell types including HepG2 through macroautophagy. The requirement for ATG7 and STX17, and co-localization between APOE and autophagosome marker LC3A that we observe in HepG2 are consistent with previous work showing APOE colocalization and co-immunoprecipitation with LC3 in hepatoma cells (Kim and Ou, 2018). While we did not find that knockdown of ATG7 caused intracellular APOE accumulation in ST14A neuron-like cells under basal nutrient-rich conditions, a variety of conditions may stimulate autophagic processes, including starvation, oxidative stress, ER stress, lipid stress and DNA damage (Peker and Gozuacik, 2020). In a recent study, quantitative proteomics of insoluble proteins revealed that APOE accumulates in the cortex of mice with either neuronal LAMP2A or ATG7 knocked down, suggesting that both proteins play a role in autophagic APOE degradation (Bourdenx et al., 2021). Cell-type- or brain-region-specific differences in which pathways degrade APOE merit further investigation.

We found that lysosomal degradation of APOE in HEK293 cells was not amplified by Rap by western blot analysis. Rap provides only partial inhibition of mTORC1 (Nyfeler et al., 2011), and mTORC2, which may also contribute to autophagic regulation, is only sensitive to Rap with prolonged (>24 h) treatment (Sarbassov et al., 2006). APOE may be targeted by a selective type of autophagy that is not activated by Rap; Rap has no effect on CMA (Finn et al., 2005) so it may not impact LAMP2A-mediated APOE degradation. APOE4 may induce the same macroautophagic pathway as Rap, preventing stimulation of further macroautophagic degradation with Rap treatment.

We also found that autophagy proteins were required for efficient internalization of APOE. It has recently been shown that ATG8s may be recruited to endosomes containing Aβ in a process called LANDO (Heckmann et al., 2019), which requires canonical autophagy proteins such as ATG7 and the non-canonical autophagy protein Rubicon. APOE and amyloid β may enter the cell using the same endocytic receptors (Verghese et al., 2013), and we found that knockdown of ATG7 or Rubicon reduced APOE internalization in HepG2 cells. Although ATG7 knockdown reduces APOE internalization, we observed higher levels of intracellular APOE upon ATG7 knockdown by western blotting, suggesting a profound impairment of APOE degradation by ATG7-dependent autophagy in HepG2 cells.

### Conclusion

Future studies will be needed to determine whether the increased APOE4 lysosomal trafficking observed in this study is protective, by promoting the sequestration and degradation of a toxic misfolded protein (Chen et al., 2011; Morrow et al., 2002), or harmful by causing lysosomal dysfunction, dysregulated autophagic flux and loss of extracellular lipid trafficking function (Poirier et al., 2014; Wu et al., 2014). Proposed novel therapeutic strategies for AD include both increasing APOE function (APOE mimetics, increasing APOE4 lipidation, small molecule APOE4 structure correctors, APOE2 gene therapy) and reducing APOE expression using antisense oligonucleotides (Vitek et al., 2015; Williams et al., 2020). It may be useful to modulate the balance between CMA and macroautophagy as an adjunct to these potential therapies. Our results suggest that both CMA and macroautophagy may play a role in APOE degradation. These two autophagic systems may compensate for one another when autophagy is dysregulated by aberrant lysosomal trafficking of APOE4. With aging CMA declines (Cuervo and Dice, 2000b); macroautophagy upregulation cannot adequately compensate for declining CMA under conditions of cellular stress, such as those produced by misfolded proteins, and may cause toxicity in neurons (Massey et al., 2006; Wu et al., 2014; Xilouri et al., 2009). Compounds that activate CMA may be protective to combat APOE4 lysosomal accumulation, slowing AD progression (Bourdenx et al., 2021).

In conclusion, we have shown that APOE can be degraded by autophagy and an LC3-associated endocytosis-like process, and that APOE4 is sequestered in enlarged endosomes and may perturb autophagic flux. Further study is needed to determine the functional implications of impaired APOE degradation and reduced autophagic flux in Alzheimer's disease and aging.

## MATERIALS AND METHODS

### Cell culture and transient transfection

Cell lines used are listed in Table S2. All cell lines were maintained in Dulbecco's modified Eagle's medium (DMEM; Corning 10-017-CV) supplemented with 10% FBS (Thermo Fisher Scientific 26140079). ST14A cells (Ehrlich et al., 2001) were cultured at 33°C and 5% CO_2_. All other cell lines were cultured at 37°C, 5% CO_2_. HEK293 cells (CRL-1573), HEK293T cells (CRL-3216) and HepG2 cells (HB-8065) were obtained from ATCC. ST14A cells were received as a gift from the laboratory of Elena Cattaneo and are described further in our previous work (Steffan et al., 2004). Cell lines were tested for mycoplasma contamination but were not recently authenticated.

For transient transfection of ST14A, HeLa and HEK293T cells, cells were forward-transfected using Lipofectamine 2000 (Thermo Fisher Scientific 11668027) 24 h later as instructed by manufacturer. HepG2 cells were reverse-transfected with siRNA using Lipofectamine RNAiMAX (Thermo Fisher Scientific 1377810) as per the manufacturer's instructions; transfection reagents were incubated in the bottom of a six-well plate and cells were plated directly on transfection mix. Medium was changed 24 h after forward or reverse transfection, and cells were harvested for western blotting at 48 h after transfection.

Bafilomycin A1 (Cayman Chemical 11038) was used at 50 nM for 4 h before harvesting cells for western blot. MG132 (Millipore Sigma 474791) was used at 50 µM, Epoxomicin (Sigma-Aldrich E3652-50UG) at 100 nM, ammonium chloride (NH_4_Cl) (Sigma-Aldrich A9434) at 20 mM, and chloroquine at 10 µM (Sigma-Aldrich C6628), and BFA at 5 μg/ml (Biolegend 420601) in media. Rapamycin (Sigma-Aldrich R8781) was used to stimulate autophagy at 10 nM for 4 h. Pitstop2 (1:1000) (Abcam) was used to inhibit clathrin-mediated endocytosis. Latrunculin A (50 μM) was used as a phagocytosis inhibitor.

### Plasmids

shLAMP2A and shATG7 were used as previously described (Thompson et al., 2009). APOE3–Myc–Flag was purchased from Origene (catalog number RC200395). APOE4–Myc–Flag and APOE2–Myc–Flag were generated through mutagenesis from APOE3–Myc–flag, APOE3–mCh–SepHluorin and APOE3–mCh–SepHluorin was generated through insertion cloning, and APOE3–mCh was generated through deletion cloning from APOE3–mCh–SepHluorin by GenScript Biotech. TMEM192-HA was purchased (Addgene 102930).

### Western blotting

Cells were harvested in lysis buffer containing 10% glycerol, 20 mM Tris-HCl pH 7.5, 137 mM NaCl, 1% NP40, 5 mM EDTA, and containing phosphatase inhibitors 2 (Millipore Sigma, P5726) (1:1000) and 3 (Millipore Sigma P0044) (1:1000), 5 mM nicotinamide (Sigma N3376), 5 mM butyric acid, 1 mM PMSF, 10 μg/ml aprotinin, 10 μg/ml leupeptin, and one Pierce protease inhibitor mini tablet (Thermo Fisher Scientific A32953) per 10 ml of lysis buffer. Lysates were sonicated and 20 μg of protein was then subjected to SDS/PAGE on a NuPage Novex 4-12% Bis-Tris precast gel (Thermo Fisher NW04125) with MOPS running buffer (Invitrogen NP0001) and transferred onto a Immobilon-FL PVDF (Millipore Sigma IPFL00010) membrane. Whole protein was quantified using the revert assay (LI-COR Biosciences 926-11016), and the membrane was blocked with Intercept (TBS) blocking buffer (LI-COR biosciences 927-60010) for 1 h. The membrane was then incubated in primary antibody overnight, washed three times with TBS with 0.1% Tween 20, and incubated for 1 h in Intercept block supplemented with 0.1% Tween-20 and near-infrared conjugated secondary antibody. Membranes were imaged on a LI-COR scanner and quantified using Empiria Software. Combined linear range was quantified on Empiria by analyzing a concentration gradient of protein (10, 20, 30 and 50 μg per lane) with Revert for each antibody. Experiments were performed at least twice with biological triplicates. Antibodies are listed in Table S3.

Mouse brain tissue used in this study was collected from mice similar to those we previously reported (Notomi et al., 2019). All mouse procedures were performed in strict accordance with the guidelines for Association for Research in Vision and Ophthalmology (ARVO) and experimental protocols were approved by the Animal Care Committee of Massachusetts Eye and Ear Infirmary. Brain tissue was prepared for western blot as follows: half brain was homogenized with 20 strokes of a potter-Elvenhjem glass tissue homogenizer in 1 ml modified RIPA buffer (50 mM Tris-HCl pH 7.4, 1% NP-40, 0.25% Na-deoxycholate, 150 mM NaCl, 1 mM EDTA) supplemented with one Pierce protease inhibitor mini tablet (Fisher Scientific A32953), 1 mM PMSF, phosphatase inhibitors 2 (Millipore Sigma, P5726) (1:1000) and 3 (Millipore Sigma P0044) (1:1000), 10 μg/ml aprotinin and 10 μg/ml leupeptin. Lysate was centrifuged at 16,000 ***g*** for 15 min, and 30 μg analyzed by western blotting.

### Incucyte live-cell imaging

ST14A (70,000 cells/ml), HepG2 (250,000 cells/ml) or HEK293 cells (300,000 cells/ml) were plated into Corning 96-well plates. Images were acquired in the plane of highest contrast using an IncuCyte Live Cell Analysis System (Sartorius). Three images per well were collected in phase-contrast, green and red fluorescence with a 20× lens, with *n*=3 wells per group. Quantitative fluorescence analysis was performed using the IncuCyte S3 software by creating mask settings that were applied to all images for a particular experiment.

### Quantitative RT-PCR

Cells were harvested and snap frozen. Cells were resuspended in RNeasy lysis buffer, and RNA was extracted using the RNeasy mini kit (Qiagen 74106). One microgram of RNA was reverse-transcribed into cDNA using qScript (VWR 101414-106). RT-qPCR was performed using SYBR green supermix (BioRad 1725124). Transcript levels were normalized to RPLPO Ct values. Primers are listed in Table S4.

### Preparation of fluorescent APOE conditioned medium

HEK293T cells were transfected as described above with APOE2, APOE3 or APOE4 tagged with mCh or mCh-SepHluorin. Medium was changed 24 h after transfection into Fluorobrite DMEM (ThermoFisher A1896701) with 10% FBS. Medium was collected 48 h later. Conditioned medium was centrifuged at 750 ***g*** for 5 min and transferred to a new tube to eliminate dead cell debris. Conditioned medium was applied to cells for internalization assays in a mixture of 1 part conditioned media to 1 part fresh Fluorobrite DMEM plus 10% FBS in order to prevent cell death.

### siRNA

Silencer siRNA was purchased from Thermo Fisher Scientific against human Rubicon, ATG7, and STX17. Custom silencer siRNA was ordered from Thermo Fisher Scientific against human LAMP2A (sense: 5′-GGCAGGAGUACUUAUUCUAGU-3′).

### LDL-pHrodo endocytosis assay

LDL conjugated to pHrodo was purchased from Thermo Fisher Scientific (L3455). LDL-pHrodo was diluted in media at a concentration of 10 µg/ml.

### Generation of stable cell lines

APOE-expressing HEK 293 stable cell lines were generated by transfecting APOE2, APOE3 or APOE4 tagged with mCh, mCh–SepHluorin or the fluorescent tags alone into HEK293 cells. HEK293 cells were chosen due to their rapid growth and ability to produce similar amounts of APOE3 and APOE4 intracellularly and in conditioned medium. Antibiotic selection was performed and clones were chosen to produce one APOE3–mCh-, APOE4–mCh-, and mCh-expressing cell line. Transfected cells were selected in 400 μg/ml of geneticin (G418 sulfate, Thermo Fisher Scientific 10131035). To isolate clonal colonies, cells were plated at 50 cells/ml onto six-well plates. Twelve colonies derived from a single cell were picked using a 10 μl pipette tip, and transferred to individual wells of a 96-well plate. Colonies were then expanded and harvested to check expression by western blotting. One colony/clone expressing similar amounts of APOE was then selected for each APOE isoform for further study. Cells were subsequently maintained in media with 200 μg/ml geneticin.

HepG2 cells for LysoIP assays were reverse transfected (Erfle et al., 2007) with TMEM192-HA, selected using media with 10 μg/ml Blasticidin, and maintained in 5 μg/ml Blasticidin.

### CellLight Lysosome–GFP transfection

HEK293 cells were plated at 300,000 cells/ml in a Greiner 96 well CellStar glass-bottom plate. After 24 h, 2.5 µl of CellLight Lysosome–GFP Bacmam 2.0 (Lamp1–GFP) was added to media to transduce cells. After a further 24 h, the medium was changed and cells were imaged live on an Olympus FV3000 microscope. Colocalization and intensity were quantified using Imaris software.

### LysoIP protocol

HepG2 cells stably expressing TMEM192–HA were plated at 250,000 cells/ml in 30 ml medium, with four replicate plates per conditioned medium group. HepG2 cells were incubated in 50% conditioned medium from HEK293 cells expressing APOE3–mCh or APOE4–mCh for 48 h. They were then incubated with 50 nmol Baf for 4 h and lysed using a dounce homogenizer. LysoIP was performed as previously described (Abu-Remaileh et al., 2017).

### Oleic acid treatment and lipid droplet staining

HEK293 cells were treated with 200 µM oleic acid (Millipore Sigma CAS 112-80-1) conjugated to BSA as previously described (Alsabeeh et al., 2018) overnight to stimulate LD formation (Brasaemle and Wolins, 2016). Neutral lipids were stained using a 2 µM solution of Bodipy 493/503 (Thermo Fisher D3922) as previously described (Qiu and Simon, 2016).

### Imaging and image analysis

Immunocytochemistry was performed as previously described (Grima et al., 2017). Antibodies are listed in Table S3. Fixed and live cells were imaged live on an Olympus FV3000 microscope with a 20× objective. Three images were collected from at least three wells per treatment or genotype.

Imaris analysis software was used to collect total intensity data for each channel. A colocalization channel was created using the same threshold for each image. Colocalization was quantified as the value of intensity of the coloc channel divided by total red or green fluorescence. Size of APOE puncta and lysosomes was quantified by creating red and green surface masks, and determining the average size of these surfaces. Ratio of green to red fluorescence area was calculated by creating a red and green surface and dividing total green voxels per image by total red voxels per image. Surface area of endosomes per cell was determined by creating a surface in the green channel and dividing total surface area for an image by the number of cells, quantified using the spots function to count DAPI positive nuclei. In Figs S1 and S8C, in order to assess colocalization of APOE spots with autophagic puncta, a ‘spots’ mask was created for APOE and for LC3A and LC3B staining, and number of spots with centers within 1 μm of one another were divided by total number of LC3A and LC3B spots per image.

### Native western blotting

Lipidation of APOE in conditioned medium was assessed using native gel analysis. 20 µl of conditioned medium and 2 μl of HA-tagged APOE protein purified from bacteria was added to 6.25 l NativePAGE 4× sample buffer (Invitrogen BN2008) and run on a NativePAGE mini gel (Invitrogen BN2112BX10) in light and dark cathode buffer and anode buffer made with NativePAGE running buffer (Invitrogen BN20001). Gel was transferred to an Immobilon-P PVDF membrane (Millipore IPVH00010). Membrane was blocked with Pierce StartingBlock Buffer (Fisher Scientific EN37543) for 30 min and incubated overnight with primary antibody. The following day, membrane was washed three times with TBS with 0.1% Tween-20, and incubated for 1 h in StartingBlock Buffer with HRP-conjugated secondary antibody. SuperSignal West Dura Chemiluminescent reagents (Fisher Scientific PI34076) were used to detect HRP, and membrane was imaged on an Azure c600 system.

### Mass spectrometry methods

Pulldown eluates were cleaned up by methanol-chloroform precipitation. Pellets were resuspended in 50 mM 50 mM triethylammonium bicarbonate (TEAB, Sigma-Aldrich), then reduced with the addition of 1 μl of 500 mM tris(2-carboxyethyl)phosphine (Thermo Fisher Scientific) and incubated at 37°C for 1 h. Following, 3 µl of 500 mM Iodoacetamide (Sigma-Aldrich) was added to each sample. Samples were incubated in the dark at room temperature for 1 h. 500 µl of 50 mM TEAB was added to the samples, along with 2 µl (400 ng) of trypsin/lysC mix (Promega, Madison, WI). Samples were digested at 37°C overnight (16 h). Sample peptide quantities were measured using a colorimetric peptide assay (Thermo Scientific), and volumes for 4 µg were loaded onto the nano-LC-MS system for analysis.

Liquid chromatography was performed on a Thermo nLC1200 in single-pump trapping mode with a Thermo PepMap RSLC C18 EASY-spray column (2 μm, 100 Å, 75 μm×25 cm) and a Pepmap C18 trap column (3 μm, 100 Å, 75 μm×20 mm). Solvents used were A, water with 0.1% formic acid and B, 80% acetonitrile with 0.1% formic acid. Samples were separated at 300 nl/min with a 250 min gradient starting at 3% B increasing to 30% B from 1 to 231 min, then to 85% B at 241 min, holding for 10 min.

Mass spectrometry data was acquired on a Thermo Orbitrap Fusion in data-dependent mode. A full scan was conducted using 60k resolution in the Orbitrap in positive mode. Precursors for tandem MS (MS^2^) were filtered by monoisotopic peak determination for peptides, intensity threshold 5.0×10^−3^, charge state 2–7, and 60 s dynamic exclusion after 1 analysis with a mass tolerance of 10 ppm. Higher-energy C-trap dissociation (HCD) spectra were collected in ion trap MS^2^ at 35% energy and isolation window 1.6 *m*/*z*.

Results were searched individually in Proteome Discoverer 2.2 (Thermo Scientific) against the UniProt FASTA database for *Homo sapiens* (UP000005640). The precursor mass tolerance was set to 10 ppm and fragment mass tolerance to 0.6 Da. Fixed modifications were carbamidomethyl (Cys +57.021 Da) and dynamic modifications included phospho (Serine, Threonine, Tyrosine +79.966 Da), methionine oxidation (+15.995 Da) N-terminal acetylation (+42.011 Da). Results were filtered to a strict 1% false discovery rate. Samples were compared by label-free quantitation, normalizing to total peptide amount. For abundance ratios between replicate groups, *P*-values calculated by individual protein ANOVA.

### Statistics

All experiments were performed twice with at least three biological triplicates (three separate cell culture wells). All qPCR experiments were performed with technical triplicates and statistics for qPCR performed on dCT values. All incucyte and confocal imaging experiments included three or four images averaged for each well. Statistics were performed using PRISM software. One-way ANOVA with multiple comparisons was corrected using Tukey's correction. For incucyte data with multiple time points, bars above graphs indicate time points at which significance for the false delivery rate (FDR) of <0.05 was found; two-way ANOVA with mixed effect model repeated measures was corrected for multiple comparisons by controlling the False Discovery Rate with the two-stage step-up method of Benjamini, Krieger and Yekutieli. For all other two-way ANOVAs, Tukey correction for multiple comparisons was used. Significance is indicated as *P<0.05, **P<0.01, ***P<0.001 and ****P<0.0001. In Fig. S7C proteomics analysis, one-way ANOVA with Tukey–Kramer test was used, significance of *P*<0.05 is indicated by colored boxes. All average values displayed are mean values, and error bars represent s.e.m. Further details including number of replicates and statistical methods for each experiment included in Table S5.

### Software

Incucyte live cell analysis by Sartorius was used to analyze images from the Incucyte live cell imaging system. Prism software by GraphPad was used for statistical analysis. Empiria software by Licor was used for western blot analysis. Imaris and FIJI were used for image analysis.

## Supplementary Material

Supplementary information

Reviewer comments
