# Supplementary material for: Isoform-dependent lysosomal degradation and internalization of apolipoprotein E requires autophagy proteins
Source: J Cell Sci. 2022 Jan 25;135(2):jcs258687. doi: 10.1242/jcs.258687 (PMC8917355; doi:10.1242/jcs.258687)
Supplement: Supplementary information [file joces-135-258687-s1.pdf]

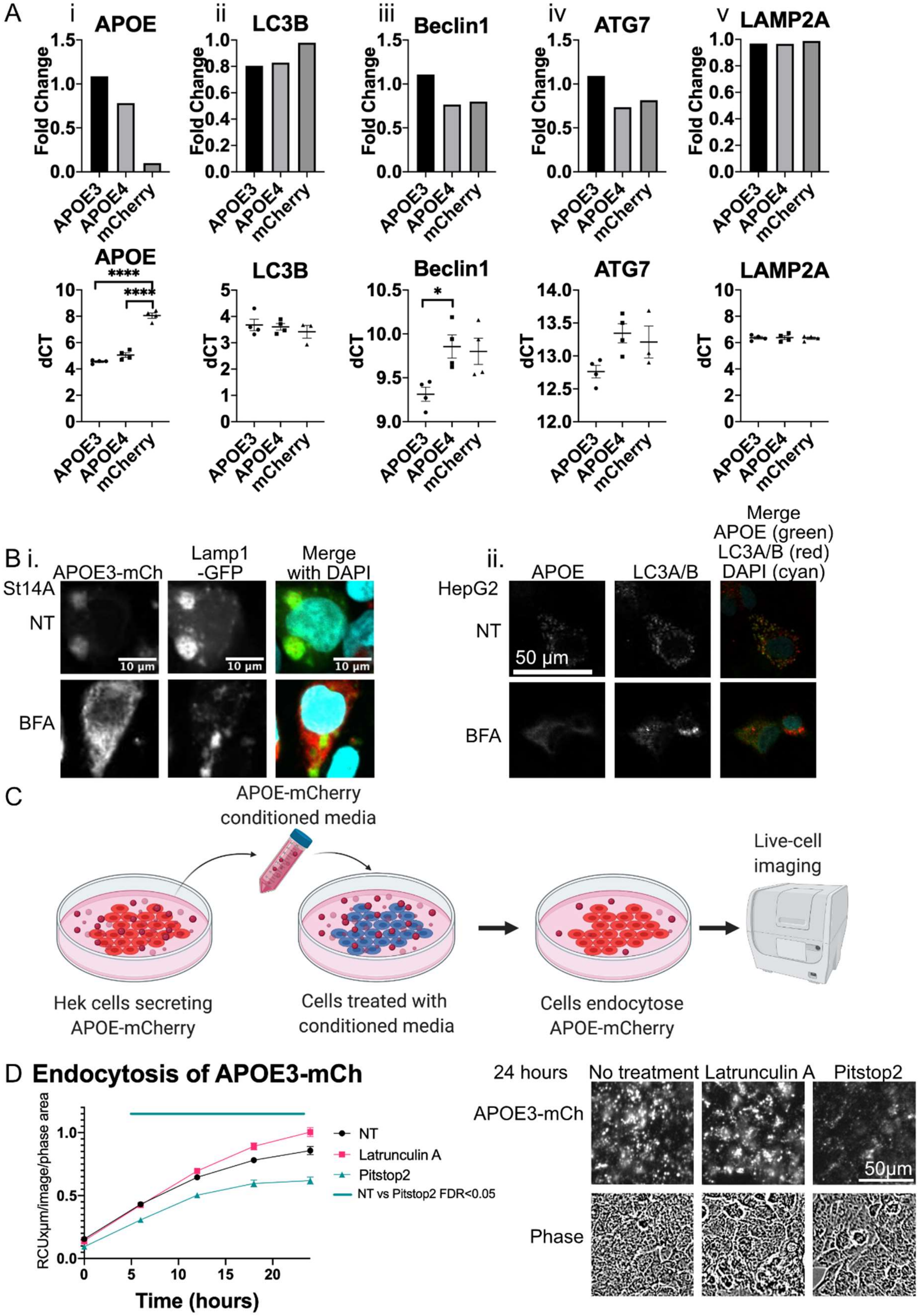
A
i APOE
ii LC3B
iii Beclin1
iv ATG7
v LAMP2A
Fold Change
dCT
APOE3
APOE4
mCherry
****
*
B i.
St14A
APOE3-mCh
Lamp1 -GFP
Merge with DAPI
NT
BFA
10 µm
ii.
HepG2
APOE
LC3A/B
Merge APOE (green) LC3A/B (red) DAPI (cyan)
50 µm
C
APOE-mCherry conditioned media
Live-cell imaging
Hek cells secreting APOE-mCherry
Cells treated with conditioned media
Cells endocytose APOE-mCherry
D Endocytosis of APOE3-mCh
RCUxµm/image/phase area
Time (hours)
NT
Latrunculin A
Pitstop2
NT vs Pitstop2 FDR<0.05
24 hours
No treatment
Latrunculin A
Pitstop2
APOE3-mCh
Phase
50µm

Fig. S1. APOE is degraded by autophagy. (**A**) 4 wells of HEK293 cells expressing APOE3 or 4-mCh were harvested for qPCR. dCT values were compared using one-way ANOVA. Ai: APOE, Aii: LC3B, Aiii: Beclin1, Aiv:ATG7, Av:LAMP2A. (**Bi**) HEK293 cells stably expressing APOE3-mCh were transfected with CellLight Lamp1-GFP and treated for 4 hours with BFA, then imaged by confocal microscopy, 3 images per well, 4 wells per treatment. **(Bii)** HepG2 cells endogenously expressing APOE3 were stained for APOE and LC3A/B, treated with BFA, then imaged by confocal microscopy, 3 images per well, 4 wells per treatment. **(C)** Conditioned media was collected from HEK293 cells stably expressing APOE-mCh. HepG2 cells were treated with this conditioned media, and imaged live using Incucyte. (D) HepG2 cells were treated with APOE-mCh conditioned media and Pitstop2 (1:1000) or Latrunculin A (50 μM) and imaged by incucyte.

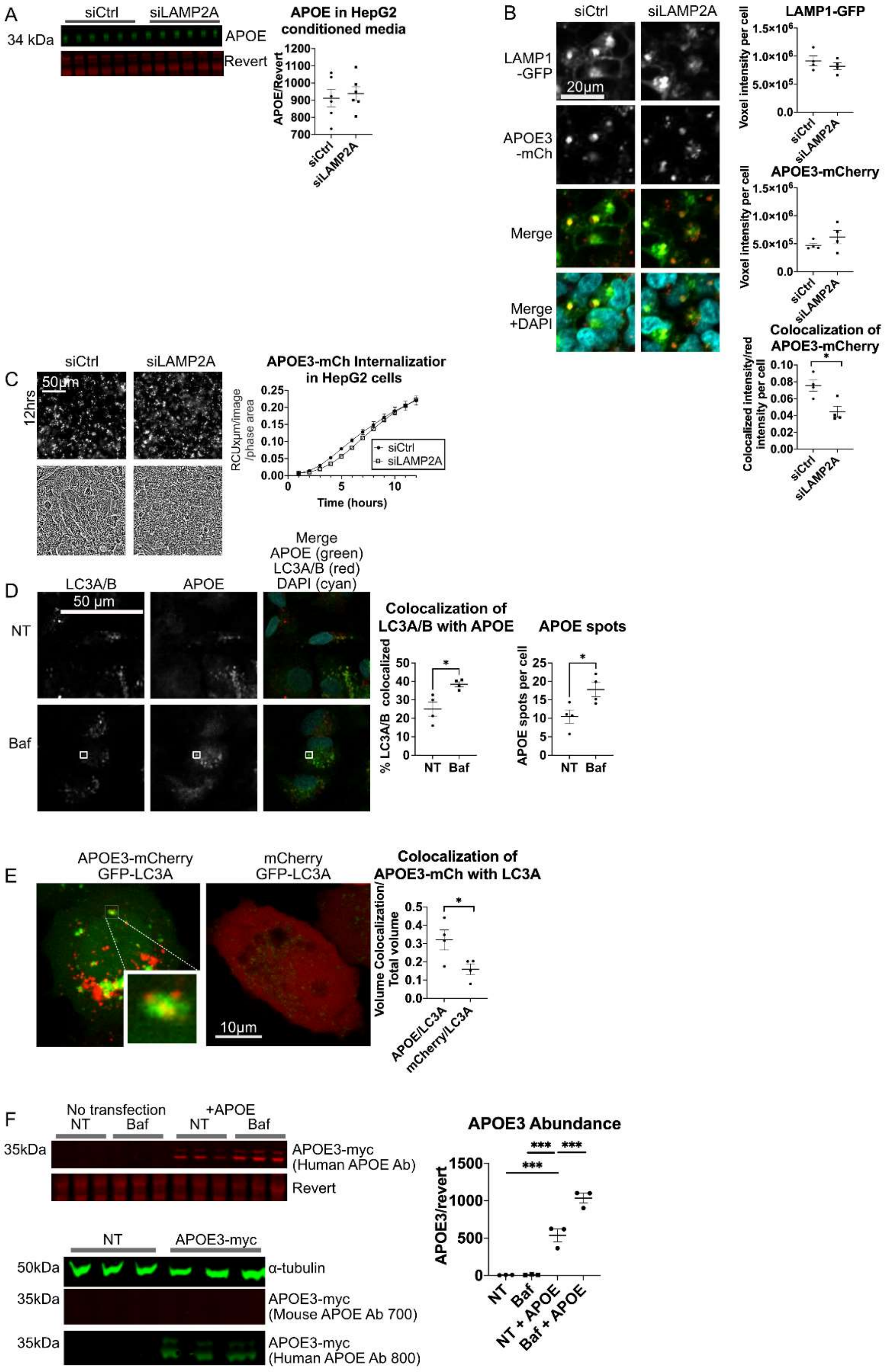

Journal of Cell Science • Supplementary information

Fig. S2. **Endogenous APOE is not detected in ST14A cells.** (**A**) Abundance of APOE in HepG2 conditioned media after transfection with siLAMP2A. Media was changed 24 hours after transfection, and conditioned media was collected 24 hours later. 15 µL of media was analyzed. (**B**) HEK293 cells expressing APOE3-mCh were transfected with Lamp1-GFP. n=3 images per well taken at 20x by confocal microscopy, 3 wells per image. (**C**) HepG2 cells transfected with siLAMP2A were treated with conditioned media from HEK293T cells secreting APOE3-mCh. Cells were imaged for 12 hours every hour and fluorescence quantified using Incucyte. Control and LAMP2A knockdown were compared by two-way repeated measures ANOVA adjusted for multiple comparisons. (D) HepG2 cells were stained for APOE and L3A/B with and without 4 hour Baf treatment. Colocalization was quantified using Imaris. (**E**) APOE3-mCh or mCh alone were co-transfected into HeLa cells with GFP-LC3A. $P<0.05$ by student's two-tailed T test. n= 4 wells per construct, 3 images per well, at least 50 cells total. Co-localization was quantified using Imaris software. (**F**) ST14A cells were transiently transfected with APOE3-myc-flag or vector. Cells were treated with or without Baf (50nM 4hrs) and lysed for western blot analysis. One-way ANOVA with Tukey correction for multiple comparisons was used to compare NT+APOE to other groups. $p<0.001$ indicated by ***. Antibody detecting mouse/rat APOE does not detect any expression in ST14A cells, and antibody against human APOE detects expression in transfected cells only.

Journal of Cell Science • Supplementary information

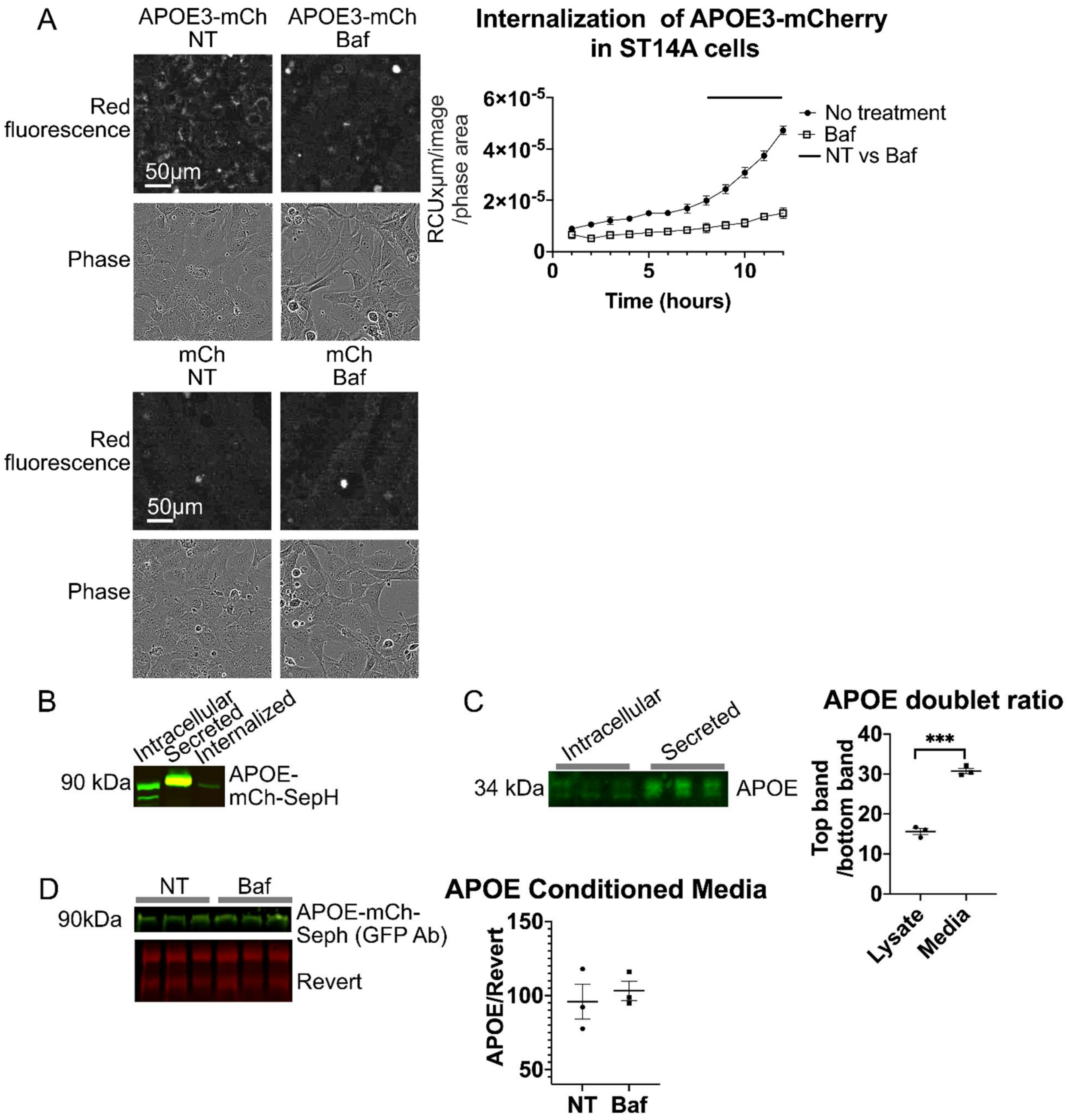
A
APOE3-mCh NT
APOE3-mCh Baf
Red fluorescence
50µm
Phase
mCh NT
mCh Baf
Red fluorescence
50µm
Phase
Internalization of APOE3-mCherry in ST14A cells
RCUxµm/image /phase area
6×10-5
4×10-5
2×10-5
0
0
5
10
Time (hours)
No treatment
Baf
NT vs Baf
B
Intracellular
Secreted
Internalized
90 kDa
APOE-mCh-SepH
C
Intracellular
Secreted
34 kDa
APOE
APOE doublet ratio
Top band /bottom band
40
30
20
10
0
***
Lysate
Media
D
NT
Baf
90kDa
APOE-mCh-Seph (GFP Ab)
Revert
APOE Conditioned Media
APOE/Revert
150
100
50
NT
Baf

Fig. S3. Bafilomycin A1 blocks internalization of APOE. (**A**) ST14A cells were cultured in conditioned media containing APOE-mCh with or without Baf. Fluorescence intensity was quantified using Incucyte Live cell analysis systems and normalized to phase confluence. Bars indicate time points at which significance was found according to two-way repeated measures ANOVA corrected for multiple comparisons at false discovery rate <0.05. (**B**) Lane labeled "intracellular" is lysate from Hek293T cells transfected with dual-tagged APOE3; lane labeled "secreted" is conditioned media from these cells. Lane labeled "endocytosed" is ST14A cell lysate following treatment with Hek293T conditioned media. Experiment performed in triplicate. Antibody against APOE was used in red (NIR 700), antibody against GFP was used in green (NIR 800). (**C**) HepG2 cell lysate (20 µg) and conditioned media (20L). (**D**) Conditioned media was collected from ST14A cells expressing APOE-mCh-SepH, with or without Baf (50nM, 4hrs). 20 µL of media was run on western blot and normalized to total protein stain.

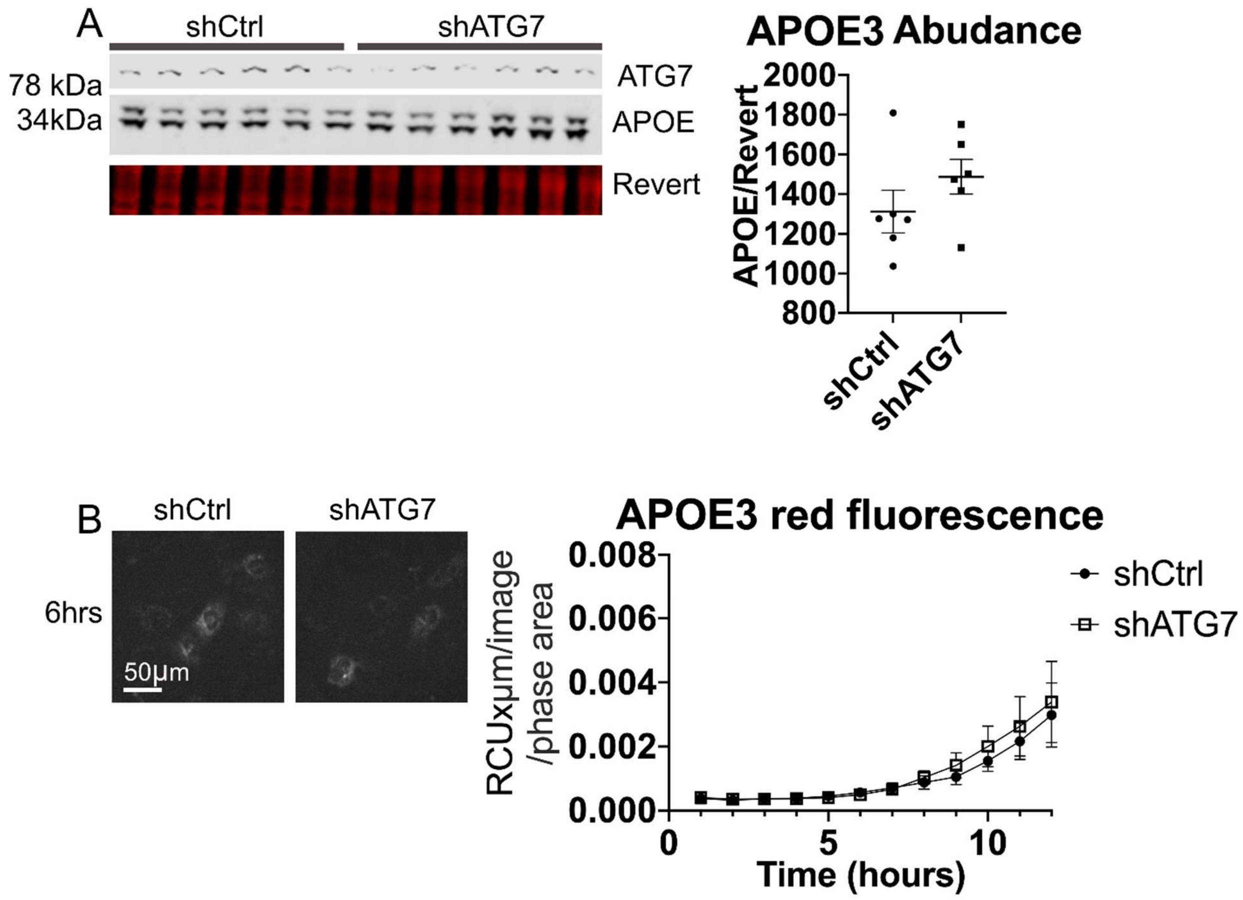

Fig. S4. Macro-autophagy does not contribute to APOE3 degradation in ST14A cells. (A-B) ST14A cells were co-transfected with APOE3 and shRNA against ATG7. APOE3 abundance was analyzed by western blot (**A**) and fluorescence quantified by Incucyte (**B**). Student's two-tailed T test was performed to analyze western blots, and two-way repeated measures ANOVA adjusted for multiple comparisons was performed to analyze fluorescent intensity of live cell images.

Journal of Cell Science • Supplementary information

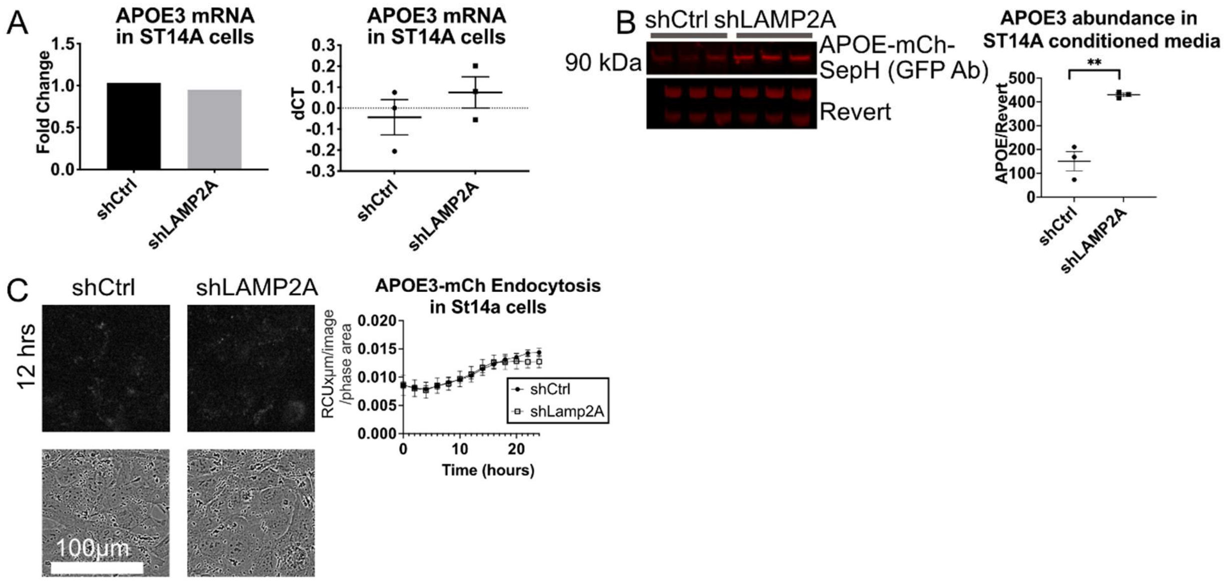

Fig. S5. LAMP2A knockdown does not affect APOE transcription, secretion, or internalization in ST14A cells. (**A**) Knockdown of LAMP2A by shRNA does not increase APOE transcript in ST14A cells. n=3 wells. (**B**) Abundance of APOE in media conditioned by ST14A cells transfected with APOE3 and LAMP2A shRNA. (**C**) ST14A cells were transfected with shLAMP2A and treated with conditioned media from HEK293T cells secreting APOE3-mCh.

Journal of Cell Science • Supplementary information

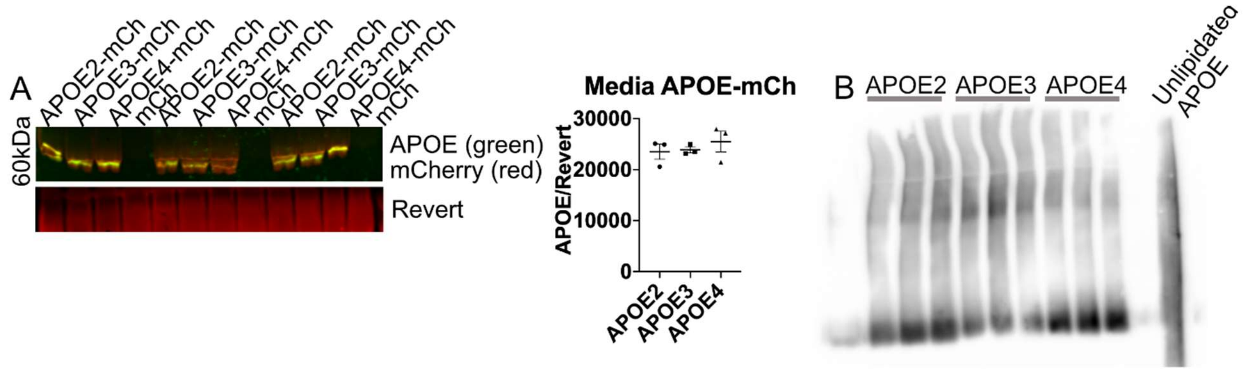

Fig. S6. Fluorescently tagged APOE is secreted by HEK293T cells. (**A**) 20μL of media conditioned by HEK293T cells transfected with APOE-mCh constructs was analyzed by western blot. One-way ANOVA analysis of APOE2, APOE3, and APOE4 levels revealed no significant difference in levels. No bands were detected in cells expressing mCh-vector, suggesting the vector is not secreted into media alone. (**B**) Conditioned media was assessed by native gel to demonstrate that APOE secreted from HEK293T cells is lipidated.

Journal of Cell Science • Supplementary information

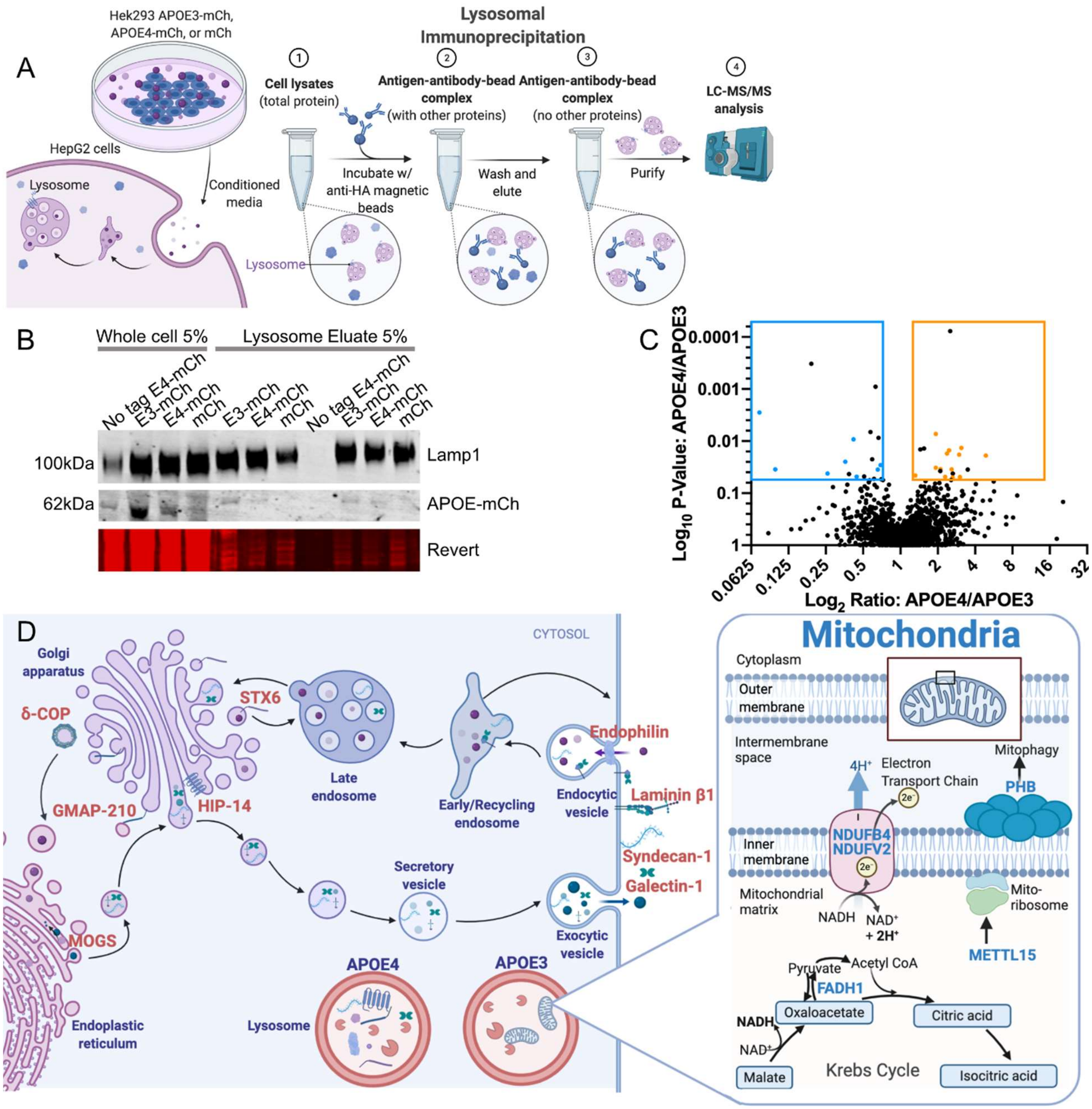

Fig. S7. Immunoprecipitation and proteomic analysis of lysosomes in cells endocytosing APOE. (**A-B**) Lysosomes were immunoprecipitated and proteomic contents analyzed by mass spectrometry from HepG2 cells treated for 24 hours with conditioned media from HEK293 cells expressing APOE3 or E4-mCh. (**C**) Proteomics of lysosomes was analyzed by ANOVA. n=4 samples per group. Orange and blue boxes indicate significantly ($p<0.05$) reduced and increased proteins in APOE4-treated samples relative to APOE3-treated samples, respectively. Proteins that were not significantly altered in the same direction as Vector relative to APOE3 are represented by colored dots. (**D**) Proteins labeled in orange indicate increase in APOE4 lysosomes relative to APOE3, blue indicates decrease in APOE4 lysosomes. Created with BioRender.com.

Journal of Cell Science • Supplementary information

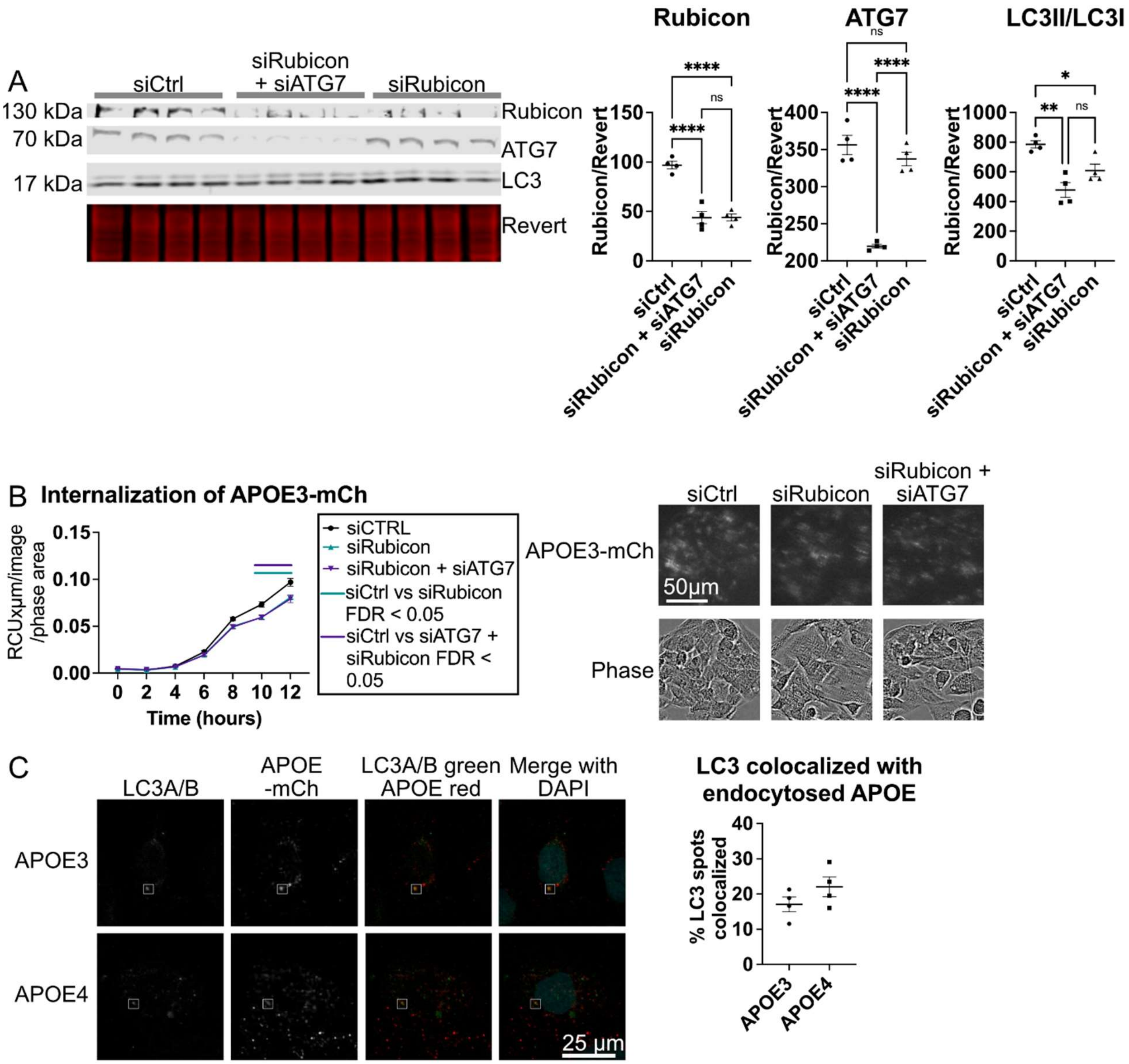

Fig. S8. Validation of Rubicon knockdown and co-knockdown of Rubicon and ATG7. (**A**) HepG2 cells were reverse transfected with siCtrl, siCtrl+ siATG7 or siRubicon, or siATG7 and siRubicon in equal amounts. Western blot was used to validate knockdown. (**B**) HepG2 siRNA transfected cells were treated with APOE3-mCh conditioned media and imaged using Incucyte. (C) ST14A cells were treated with APOE-mCh conditioned media for 24 hrs and co-stained with mCh and LC3A/B, imaged by confocal microscopy and analyzed using Imaris.

Journal of Cell Science • Supplementary information

Table S1. LysoIP Proteomics Results.

Accession	Description	Abundance Ratio: (APOE4) / (APOE3)	Abundance Ratio: (Vector) / (APOE3)	Abundance Ratio: (Vector) / (APOE4)	Abundance Ratio P-Value: (APOE4) / (APOE3)	Abundance Ratio P-Value: (Vector) / (APOE3)	Abundance Ratio P-Value: (Vector) / (APOE4)
O95302	FKBP9	0.073	0.871	11.986	0.002824986	0.915019508	0.003497797
A6NJ78	METTL15	0.098	0.16	1.64	0.03482823	0.191564201	0.416755153
O95168	NDUFB4	0.258	0.956	3.701	0.04188126		
P55036	PSMD4	0.359	0.442	1.233	0.024837183	0.071143369	0.849642682
Q6P587	FAHD1	0.418	2.239	5.356	0.00922607	0.0404347	0.001189686
Q969V3	NCLN	0.443	0.551	1.242	0.048365929	0.267834332	0.51366258
E7EPT4	NDUFV2	0.657	1.452	2.212	0.035113201	0.694864927	0.01111817
P35232	PHB	0.692	1.216	1.757	0.028869173	0.58957946	0.006270202
P18827	SDC1	1.315	0.01	0.01	0.045668619		
P28066	PSMA5	1.926	1.848	0.96	0.032426471	0.080605384	0.831370351
P46782	RPS5	1.929	1.222	0.634	0.00726678	0.680894178	0.026505119
J3KTA4	DDX5	2.103	0.819	0.389	0.034558926	0.483428179	0.005640004
P09382	LGALS1	2.291	0.93	0.406	0.048483133	0.960807773	0.052606157
Q15643	GMAP-210	2.375	1.406	0.592	0.01726162	0.55353193	0.034916173
O43752	STX6	2.466	1.75	0.709	0.014994447	0.182800753	0.361533823
B0YIW6	ARCN1	2.593	2.2	0.849	0.03498909	0.096259352	0.7962403
Q7Z5L9	IRF2BP2	2.636	0.517	0.196	0.049868561	0.095142061	0.001160372
G3XAI2	LAMB1	2.645	2.04	0.771	0.041885676	0.219122637	0.642624196
P62269	RSP18	2.973	0.776	0.261	0.018197747	0.972097635	0.012876609
Q13724	MOGS	3.016	2.47	0.819	0.049048219	0.08416334	0.936049822
Q99961	SH3GL1	3.104	1.665	0.536	0.013556205	0.25573337	0.096286662
Q8IUH5	ZDHHC17	4.889	0.01	0.01	0.019007889	ND	ND

Journal of Cell Science • Supplementary information

Table S2. Cell lines and APOE expression.

Name	Cell type	APOE expression	Rationale
HepG2	Immortalized human hepatic cells	Endogenous APOE expression	Previous work has shown lysosomal degradation of APOE in hepatic cells
ST14A	Immortalized Rat neuronal cells	Transient transfection	Neuron-like immortalized cells may have increased relevance to neurodegenerative disease, morphology amenable to imaging, high transfection efficiency
HEK 293T	Human Embryonic Kidney cells	Transient transfection	Produce high yield and equal amounts of APOE2, APOE3, and APOE4 in conditioned media
HEK 293	Human Embryonic Kidney cells	Stable expression via transfection and clonal antibiotic selection	Avoid stress of transfection reagents, avoid fluctuation of expression over time, avoid variability in transfection across isoforms

Journal of Cell Science • Supplementary information

Table S3. Antibodies.

Target	Company and Catalogue number	Application, concentration
APOE	Biolegend 803301	Western blot 1:500
mCherry	Biolegend 677702	Western blot 1:500
P62/SQSTM1	Abnova PAB16850	Western blot 1:1000
LAMP2A	Boster M01573	Western blot 1:500
LAMP2A	ThermoFisher Scientific # 51-2200	Western blot 1:1000
Beclin1	Boster Bio PB9076	Western blot 1:1000
Mouse APOE	Abcam ab183596	Western blot 1:1000
APOE	Invitrogen 701241	Western blot 1:500
GAPDH	Invitrogen MA5-15738	Western blot 1:1000
VPS35	Boster Bio M01644	Western blot 1:500
Rab7	Boster Bio PB9883	Immunocytochemistry 1:200
Flag/DDK	Origene TA180144	Western blot 1:1000
LC3A/B	Cell Signaling	Western blot 1:1000
EEA1	Cell Signaling 2411S	Immunocytochemistry 1:200
HA	Sigma H6908	Western blot 1:1000
α-tubulin	Sigma T6074-200	Western blot 1:2000
ATG7	Millipore Sigma A2856	Western blot 1:1000
GFP	Takara 632569	Western blot 1:1000
STX17	Proteintech 17815-1-AP	Western blot 1:500
Secondary ms HRP	Jackson Immuno Research 115035146	Western blot 1:2000
Secondary NIR 800 ms	Licor 92632210	
Secondary NIR 700 Rb	Licor 92632211	Western blot 1:2000

Table S4. qPCR primers.

Target	Species	Sequence (F)	Sequence (R)
LAMP2A	Rat	GGCAATGCTACCTGTCTGCTGGC	AGAATAAGTACTCCTCCCAGGGCTGC
RPLPO	Rat	TGATCATCCAGCAGGTGTTTGA	ACAGACAAAGCCAGGACCCTTT
APOE	Human	GGGTCGCTTTTGGGATTACCTG	CAACTCCTTCATGGTCTCGTCC
RPLPO	Human	TGGTCATCCAGCAGGTGTTCGA	ACAGACACTGGCAACATTGCGG
LAMP2A	Human	GGCAATGATACTTGTCTGCTGGC	GTAGAGCAGTGTGAGAACGGCA
LAMP2B	Human	GAAGGAAGTGAACATCAGCATG	CAAGCCTGAAAGACCAGCACC
Rubicon	Human	CGACCTGGAAAAGGAGAATGCC	TCCTCTAGGCACTGGCTCATCA
Beclin1	Human	CTGGACACTCAGCTCAACGTCA	CTCTAGTGCCAGCTCCTTTAGC
ATG7	Human	CGTTGCCCACAGCATCATCTTC	CACTGAGGTTCACCATCCTTGG
LC3B	Human	GAGAAGCAGCTTCCTGTTCTGG	GTGTCCGTTCACCAACAGGAAG
STX17	Human	TCCATGACTGTTGGTGGAGCA	CAGTGCAATTCCTGCACTT

Table S5. Statistical analyses. All qPCR experiments were performed with technical triplicates and statistics for qPCR performed on dCT values. All incucyte experiments included 3 or 4 images averaged for each well. T test: Two-tailed student's T test; significance: $p<0.05$ *, $p<0.01$ **, $p<0.001$ ***, $p<0.0001$ ****. One way ANOVA 1: One-way ANOVA with multiple comparisons correction; significance: $p<0.05$ *, $p<0.01$ **, $P<0.001$ ***, $p<0.0001$ ****. One way ANOVA 2: One-way ANOVA; significance: $p<0.05$ indicated by colored boxes. Two way ANOVA 1: Two-way mixed effects repeated measures ANOVA corrected for multiple comparisons with false discovery rate; Bars above graphs indicate time points at which significance was found. Significance: FDR <0.05. Two way ANOVA 2: Two-way ANOVA with Tukey correction for multiple comparisons. Significance: $p<0.05$ *, $p<0.01$ **, $p<0.001$ ***, $p<0.0001$ ****.

Figure	Replicates	Statistical test
1A	4 wells per genotype	One-way ANOVA 1
1B	3 wells per treatment	One-way ANOVA 1
1C	4 wells per genotype	One-way ANOVA 1
2	4 wells per treatment	Two-way ANOVA 1
3A-B	3 wells per genotype, 3 images per well	T test
3C	4 wells per treatment	Two-way ANOVA 2
4 A-F, H, I	6 wells per treatment or siRNA	T test
4G	6 wells per treatment	T test
4J	4 wells per siRNA	One-way ANOVA 1
5B, E	4 wells per genotype and treatment	Two-way ANOVA 1
5C	3 wells per treatment	T test
5D	3 wells per treatment	T test
5F	6 wells per treatment	T test
6A	6 wells per genotype/ treatment	T test
6B	4 wells per genotype and treatment	Two-way ANOVA 1
6C	3 animals per genotype	T test
7A	5 wells per conditioned media genotype	Two-way ANOVA 1
7B	3 wells per conditioned media genotype	Two-way ANOVA 1
7C	3 wells per genotype	Two-way ANOVA 2
7D	4 wells per conditioned media genotype	One-way ANOVA 1
8A-B	6 wells per siRNA	Two-way ANOVA 1
8C	6 wells per siRNA	T test
8D	4 wells per treatment	T test
8E	5 wells per treatment	Two-way ANOVA 1
S1A	4 wells per genotype	One-way ANOVA 1

Journal of Cell Science • Supplementary information

S1B	3 wells per treatment	No statistics
S1D	3 wells per treatment	Two-way ANOVA 1
S2A	6 wells per siRNA	T test
S2B	4 wells per siRNA	T test
S2C	6 wells per siRNA	Two-way ANOVA 1
S2D	4 wells per treatment	T test
S1E	4 wells per APOE3-mCh vs mCh transfection	T test
S2F	3 wells per transfection and treatment	One-way ANOVA 1
S3A	3 wells per conditioned media treatment	Two-way ANOVA 1
S3C-D	3 wells per treatment	T test
S4A	6 wells per genotype	T test
S4B	3 wells per shRNA or treatment	Two-way ANOVA 1
S5A	3 wells per genotype	T test
S5B	3 wells per shRNA	T test
S5C	6 wells per shRNA	Two-way ANOVA 1
S6A	3 wells per conditioned media genotype	One-way ANOVA 1
S7C	4 wells per conditioned media genotype	One-way ANOVA 2
S8A	4 wells per siRNA	One-way ANOVA 1
S8B	5 wells per siRNA	Two-way ANOVA 1
S8C	4 wells per conditioned media genotype, 4 images per well	T test

Journal of Cell Science • Supplementary information

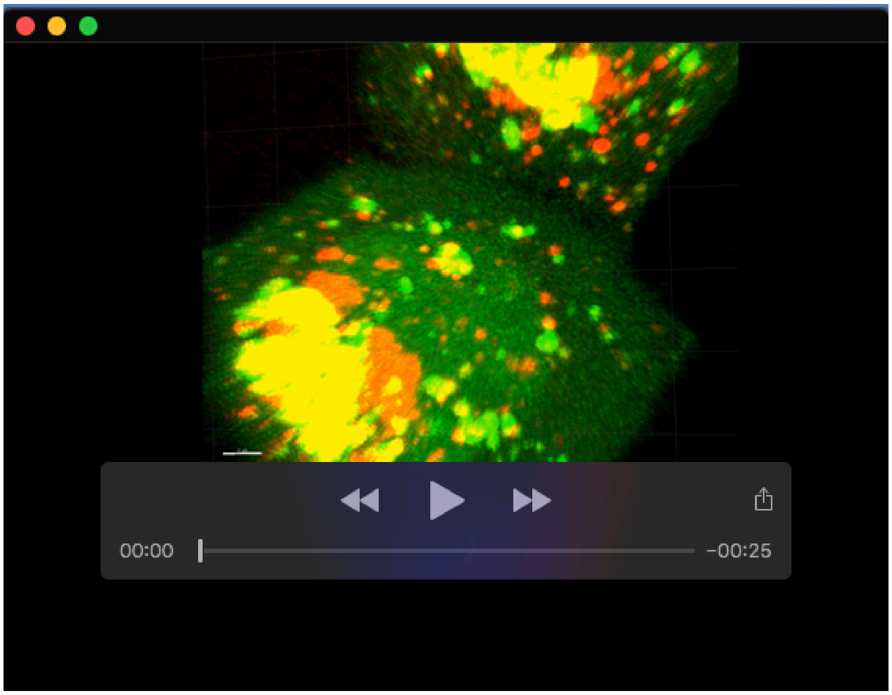

Movie 1. APOE3-mCh is contained in LC3A-positive vesicles. APOE3-mCh was co-transfected into HeLa cells with GFP-LC3A. Quantification of colocalization shown in Fig. S2E. 3-dimensional analysis was performed using Imaris software.

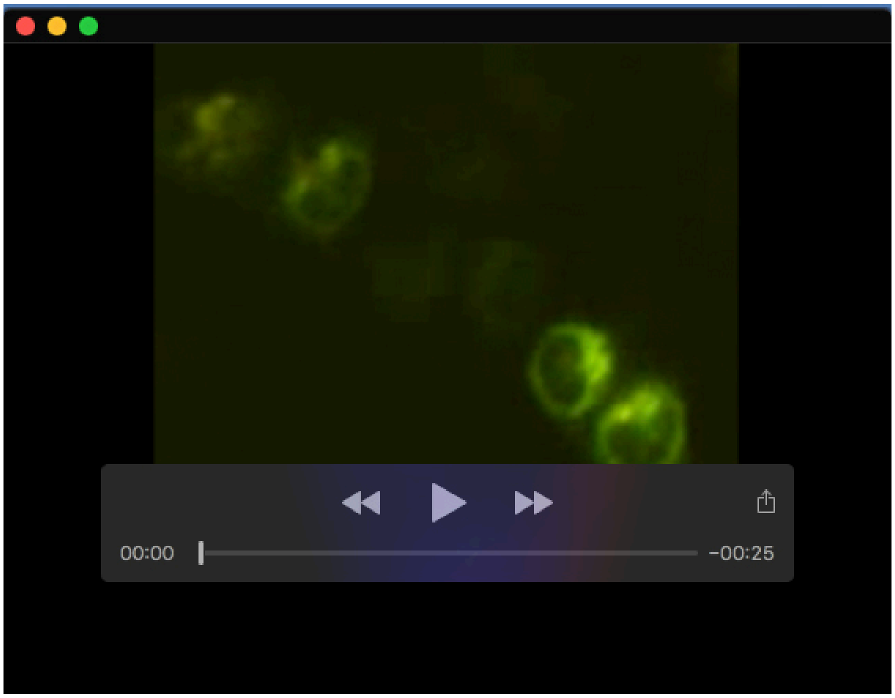

Movie 2. Dual-tagged APOE3 transiently over-expressed in ST14A cells. Dual-tag fluorescent APOE with quenching of green SepHluorin over time as APOE3 enters acidic compartments. Imaging was performed using Incucyte live cell analysis. Quantification in Fig. 5B.

Journal of Cell Science • Supplementary information
