# Supplementary material for: Isoform-dependent lysosomal degradation and internalization of apolipoprotein E requires autophagy proteins
Source: J Cell Sci. 2022 Jan 25;135(2):jcs258687. doi: 10.1242/jcs.258687 (PMC8917355; doi:10.1242/jcs.258687)
Supplement: Reviewer comments [file joces-135-258687_review_history.pdf]

Isoform-dependent lysosomal degradation and internalization of apolipoprotein E requires autophagy proteins

Gianna M. Fote, Nicolette R. Geller, Nikolaos E. Efstathiou, Nathan Hendricks, Demetrios G. Vavvas, Jack C. Reidling, Leslie M. Thompson and Joan S. Steffan
DOI: 10.1242/jcs.258687

Editor: Jennifer Lippincott-Schwartz

Review timeline

Original submission:	24 March 2021
Editorial decision:	29 April 2021
First revision received:	29 October 2021
Accepted:	17 December 2021

Original submission

First decision letter

MS ID#: JOCES/2021/258687

MS TITLE: Isoform-dependent lysosomal degradation and internalization of Apolipoprotein E requires autophagy proteins

AUTHORS: Gianna M. Fote, Nicolette R. Geller, Nikolaos Efstathiou, Nathan Hendricks, Demetrios G. Vavvas, Jack C. Reidling, Leslie M. Thompson, and Joan S. Steffan
ARTICLE TYPE: Research Article

We have now reached a decision on the above manuscript.

To see the reviewers' reports and a copy of this decision letter, please go to: https://submit-jcs.biologists.org and click on the 'Manuscripts with Decisions' queue in the Author Area. (Corresponding author only has access to reviews.)

As you will see, the reviewers raise a number of substantial criticisms that prevent me from accepting the paper at this stage. They suggest, however, that a revised version might prove acceptable, if you can address their concerns. If you think that you can deal satisfactorily with the criticisms on revision, I would be pleased to see a revised manuscript. We would then return it to the reviewers.

We are aware that you may be experiencing disruption to the normal running of your lab that makes experimental revisions challenging. If it would be helpful, we encourage you to contact us to discuss your revision in greater detail. Please send us a point-by-point response indicating where you are able to address concerns raised (either experimentally or by changes to the text) and where you will not be able to do so within the normal timeframe of a revision. We will then provide further guidance. Please also note that we are happy to extend revision timeframes as necessary.

Please ensure that you clearly highlight all changes made in the revised manuscript. Please avoid using 'Tracked changes' in Word files as these are lost in PDF conversion.

© 2022. Published by The Company of Biologists under the terms of the Creative Commons Attribution License (https://creativecommons.org/licenses/by/4.0/).

I should be grateful if you would also provide a point-by-point response detailing how you have dealt with the points raised by the reviewers in the 'Response to Reviewers' box. Please attend to all of the reviewers' comments. If you do not agree with any of their criticisms or suggestions please explain clearly why this is so.

Reviewer 1

Advance summary and potential significance to field

APOE4 is the strongest genetic risk factor for late-onset Alzheimer’s disease, yet little is known regarding how ApoE protein is intracellularly degraded. Previous work has demonstrated that ApoE is degraded in the lysosome, and that lysosomal accumulation of ApoE4 causes swollen lysosomes and defects in proteostasis.
Here, Fote et al demonstrate that ApoE can be degraded by autophagy after leaving the Golgi. Specifically the role of LAMP2A-mediated autophagy is demonstrated using multiple cell lines, which is a novel observation in the field. ApoE4 is shown to cause impaired autophagic flux and enlarged lysosomes. E4 was also found to have a slower rate of uptake through endocytosis than E3.

All experiments presented in this paper were well performed and presented, and quantification and statistical analyses are rigorous and transparent. Key experiments are replicated in multiple cell lines using different strategies for ApoE expression, strengthening support for the paper’s findings. The observation that ApoE is degraded by LAMP2A-mediated autophagy post-Golgi is a novel result in the field and important to our understanding of ApoE protein function.

Comments for the author

Unfortunately, the paper overall lacks a clear narrative thread connecting the results of the first few figures to later ones. Figures 1, 3, and 4 begin to build an interesting story uncovering how ApoE is degraded, but these novel results are not well developed. The mechanism of this paper’s novel observation, LAMP2A-mediated degradation of ApoE, should be further explored. One key aspect that remains unclear regarding the autophagic degradation of ApoE is whether this degradation occurs at the post-Golgi compartment prior to secretion, or after ApoE is secreted and then taken up by the same cell via endocytosis. The current data cannot distinguish between these possibilities. Therefore, the current claim that “ApoE from the post-Golgi compartment is degraded by autophagy” is not well supported. It is important to perform additional experiments to distinguish between these two possibilities, because the implications are very different. To distinguish between autophagy from the post-Golgi vs. after secretion/endocytosis, the authors could use inhibitors of endocytosis such as Dynasore/Pitstop. Alternatively, they could use a strategy where they photohighlight a pool of ApoE in the Golgi (e.g., by photoactivation or photoconversion) and then follow its trafficking to the lysosome. Without these types of experiments, the authors cannot make conclusions about post-Golgi trafficking. In addition, the authors suggest that ApoE may be degraded by multiple autophagic pathways, including chaperone-mediated autophagy (CMA). But if ApoE is degraded by chaperone-mediated autophagy post-Golgi, then there is currently a significant gap in the mechanism: how does ApoE escape from the secretory pathway into the cytoplasm, since CMA works on cytoplasmic proteins?
Similarly, the degradation of internalized ApoE via LC3-associated autophagy (LANDO) is poorly developed.
The authors point out that their results showing reduced internalization of ApoE upon knockdown of Rubicon or ATG7 in Figure 2 could be due to reduced endocytosis caused by impaired receptor recycling over time.
The main evidence presented to rule this out is visualization of GFP-LC3A and ApoE3-mCh in Figure 2F.
However, these images are not convincing, and no quantification of co-localization is included.
Another major point is that the authors switch between 3 different cell lines: HepG2, ST14A, and HEK293.
The ST14A immortalized neuron line is appropriate for studying internalization of ApoE. However, neurons do not typically express high levels of ApoE (indeed, the authors could not detect endogenous ApoE in ST14A cells), so this does not seem like an appropriate choice for studying post-Golgi trafficking of ApoE. For future experiments, I recommend continuing to use HepG2 cells

© 2022. Published by The Company of Biologists under the terms of the Creative Commons Attribution License (https://creativecommons.org/licenses/by/4.0/).

to study post-Golgi trafficking of ApoE, and/or switching to a cell line that better represents the cells that predominantly express and secrete ApoE in the brain (i.e., astrocytes and microglia).
In summary, the current manuscript contains interesting data about the turnover of ApoE, which is likely to be of value to the community. Unfortunately, neither of the two main observations (post-Golgi autophagy of ApoE by LAMP2-dependent autophagy and internalization of ApoE via LANDO) are well developed. Therefore my opinion is that this manuscript will require major experimental work and restructuring in order to appropriate for publication in JCS.

Reviewer 2

Advance summary and potential significance to field

In this submission by Fote and colleagues, the authors examine the degradation of the gene product of APOE, the major lipoprotein in the CNS, for which the presence of the APOE4 allele is one of the best known risk factors for late onset Alzheimer's disease. Beginning with the premise that autophagosomes and enlarged endosomes accumulate in AD brain tissue, and especially for APOE4 carriers, the team investigates if APOE might be degraded by the lysosomal pathway autophagy or through other LC3- associated processes. The authors use several immortalized cell lines, and brain tissue to identify which autophagy-related genes might be required to allow for APOE turnover. They find that a series of transcripts associated with autophagic and LC3-associated processes might affect APOE turnover, and several findings coalesce across multiple cell types, which is a strength. However, since the continuity of experiments across cell types is limited, it is difficult to determine the overall conclusion of which if any autophagy pathway might be playing a role. Moreover, there are experiments that could be interpreted as contradictory, making it uncertain how to interpret the different findings across the different cell types, since it is unclear if these events are unique to certain cell types or not. This study addresses an understudied area, and the data suggest that there might be an isoform specific impact on/relationship with lysosomal degradation. Nonetheless in its current form, while there is a lot of different kinds of data presented, the study also doesn't quite mechanistically establish what underlies this difference, which may in part be improved by providing greater cohesion between the multiple data sets.

Comments for the author

Although the comments below can help, a restructuring of the paper might also be helpful.

Overall comments
1. The presentation of the imaging data can be vastly improved, with grayscale images replacing the colored images.
2. Some of the statements were not quite accurate, and have been addressed in the comments below.
Figure 1 and related supplementary data:
1. The 'positive' data is placed in the figures, whereas the 'negative' data is placed in supplemental. To give a better picture of the data, it would be useful to show these data together. For example, LAMP2A and 2B should be shown together in the figures.
2. What is the outcome of Stx17 KD alone?
3. Please use endogenous LC3 immunofluorescence for co-localization 4. Western blots examining protein levels (whenever possible) are important to ensure that functional changes are likely 5. Related text: line 170, given the hypothesis proposed for Figure 2, LC3-positive vesicle might be more appropriate.
Figure 2 and related supplementary data:
1. The loss of Rubicon may also augment macroautophagy, hypothetically thereby driving degradation to occur faster (given the conclusion drawn at the end of Figure 1- see comment 5 above). Lysosome-inhibition to prevent degradation could be helpful here.
2. See fig 1 comment 4.
3. See Fig 1 comment 3.
4. What is the role of other atg8 orthologs in the process? Given the previously described role of GABARAPs, this might be relevant here.
5. Quantification is lacking for image based analyses (ex. Fig 2F).
Figure 3 and related supplementary data

© 2022. Published by The Company of Biologists under the terms of the Creative Commons Attribution License (https://creativecommons.org/licenses/by/4.0/).

1. See comment Fig 2 #5. Images in 3B also look over saturated.
2. Associated text line 203: the dual tag does not necessarily indicate direct trafficking. Only that the tagged protein arrives to an acidified environment.

Figure 4 and related supplementary data
1. Is there any correlation between level of KD and APOE3 levels (Fig 4A)?
2. Lamp2B should also be revisited.
3. Although the addition of the LAMP2 KO mice is nice, given that it does not segregate between the different isoforms, it is difficult to make an isoform specific conclusion, which seems critical for the conclusion here.

Figure 5 and related supplementary data
1. It is difficult to ascertain where the work is going- the association of LAMP2A with CMA is strong, and this has not been completely ruled out by the previous experiments. As for macroautophagy, the lack of a rapamycin induced event is unexpected, since cargo that get degraded in an adaptor protein-dependent manner is also usually impacted by mTOR inhibition. It is also unclear what exactly is the cargo being captured, if macroautophagy is the mechanism as implied by this section. Is it APOE or a vesicle containing APOE?
2. These experiments raise the need for understanding what type of vesicle is being examined when APOE and LC3 co-localize, and accumulate upon Baf treatment. Immunofluorescent staining for rab proteins might be helpful here.
3. 3MA would impact all membrane trafficking events for which PI3KC3 would be present, suggesting that the concentration of 3MA might not have been sufficient (for which this compound is notorious). No control is run to determine this.

Figure 6: The rationale for suddenly adding APOE4 is lacking. Why was this not examined earlier?

Figure 7 and related supplementary data:
1. Line 321: should be Fig. 7A and 7B 2. The presence of absence of Baf on LC3 levels can be assessed to see if further inhibition can be assessed.
3. How these observation are influenced by the findings in Fig. 1, and the role of rbcn is unclear. The loss of Rbcn impeded packaging of APOE, and thus if packaging is central to its degradation, it is difficult to interpret these findings.

Figure 8 and related supplementary data:
1. Although Rab7 was explored here, whether it would also be present for Figure 5 is unclear.
2. The quality of the imaging for 8C should be improved, expecially for the conclusions drawn.
3. Proteomic analyses- the analyses were done through non quantitative approaches. Thus the similarity across different runs (Pearson’s correlations)
should be to determine variability of runs. It is unclear whether corrections for multiple comparisons have been made Minor comments:
Line 176: PI3KC3 ‘usual function’ is not accurate. Rubicon can act to change in which complex PI3KC3 can reside.

First revision

<u>Author response to reviewers' comments</u>

Response to Reviewers, Fote et al. JCS resubmission:

Reviewer 1 Advance summary and potential significance to field
APOE4 is the strongest genetic risk factor for late-onset Alzheimer’s disease, yet little is known regarding how ApoE protein is intracellularly degraded. Previous work has demonstrated that ApoE is degraded in the lysosome, and that lysosomal accumulation of ApoE4 causes swollen lysosomes and defects in proteostasis. Here, Fote et al demonstrate that ApoE can be degraded by autophagy after leaving the Golgi. Specifically, the role of LAMP2A-mediated autophagy is demonstrated using multiple cell lines, which is a novel observation in the field. ApoE4 is shown to cause impaired autophagic flux and enlarged lysosomes. E4 was also found to have a slower rate of uptake through endocytosis than E3.

All experiments presented in this paper were well performed and presented, and quantification and statistical analyses are rigorous and transparent. Key experiments are replicated in multiple cell

© 2022. Published by The Company of Biologists under the terms of the Creative Commons Attribution License (https://creativecommons.org/licenses/by/4.0/).

lines using different strategies for ApoE expression, strengthening support for the paper's findings. The observation that ApoE is degraded by LAMP2A-mediated autophagy post-Golgi is a novel result in the field and important to our understanding of ApoE protein function.

We thank Reviewer 1 for noting the novelty of our results and the rigor of our methods.

Reviewer 1 Comments for the author
Unfortunately, the paper overall lacks a clear narrative thread connecting the results of the first few figures to later ones. Figures 1, 3, and 4 begin to build an interesting story uncovering how ApoE is degraded, but these novel results are not well developed.

In order to improve the continuity and narrative thread of the story, the figures and text have been rearranged to first show the differences in the autophagic degradation of the disease-associated isoform APOE4 (previously figures 5-7) followed by more mechanistic details regarding proteins involved in the autophagy of APOE (previously figures 1, 3, and 4), and finally figures having to do with the autophagy-protein dependent internalization of APOE (figures 2 and 8). We have rearranged and rewritten parts of the introduction, results, and discussion accordingly.

The mechanism of this paper's novel observation, LAMP2A-mediated degradation of ApoE, should be further explored. One key aspect that remains unclear regarding the autophagic degradation of ApoE is whether this degradation occurs at the post-Golgi compartment prior to secretion, or after ApoE is secreted and then taken up by the same cell via endocytosis. The current data cannot distinguish between these possibilities. Therefore, the current claim that "ApoE from the post-Golgi compartment is degraded by autophagy" is not well supported. It is important to perform additional experiments to distinguish between these two possibilities, because the implications are very different. To distinguish between autophagy from the post-Golgi vs. after secretion/endocytosis, the authors could use inhibitors of endocytosis such as Dynasore/Pitstop.

We thank reviewer 1 for this excellent experimental idea. We have added Figure 4C (previously Figure 1) and supplemental figure 1Bii, and the following associated text, lines 239-254: "APOE3-mCh forms puncta that co-localize with Lamp1-GFP, but with BFA treatment it loses its punctate pattern and is dispersed throughout the cell body (Fig. S 1Bi). This dispersed pattern was also observed in HepG2 cells endogenously expressing APOE that were treated with BFA (Fig. S1Bii). In order to determine whether some APOE enters the lysosome directly from the post-golgi intracellular compartment, rather than being secreted and endocytosed, cells were treated with both Baf and Pitstop 2, an inhibitor of clathrin-mediated endocytosis. In order to validate that Pitstop2 effectively inhibits endocytosis of APOE, HepG2 cells were treated with APOE3-mCherry (APOE3-mCh)-containing media from transfected HEK293T cells, which were selected based on their quick growth and consistently high yield of secreted APOE3-mCh following transfections (Fig. S 1C). APOE3-mCh that enters HepG2 cells from conditioned media is fluorescent red in the plane of greatest phase contrast (Fig. S 1D). Pitstop significantly inhibited endocytosis of APOE3-mCherry from conditioned media (Fig. S 1D), whereas Latrunculin A, which inhibits phagocytosis, had no effect. Even when endocytosis was inhibited by Pitstop 2, Baf significantly increased APOE levels (Fig. 4C), suggesting that APOE can reach the lysosome from the intracellular post-golgi compartment instead of secretion."

Alternatively, they could use a strategy where they photohighlight a pool of ApoE in the Golgi (e.g., by photoactivation or photoconversion) and then follow its trafficking to the lysosome. Without these types of experiments, the authors cannot make conclusions about post-Golgi trafficking.

We thank reviewer 1 for this very intriguing experimental idea. We consulted with the Laboratory for Fluorescence Dynamics at UCI, including experts in photoconversion methodologies and techniques. After much consideration, they advised that developing APOE constructs that can be photo-highlighted and a system where the golgi could be fluorescently marked in the desired cell types would require a year or more, and thus is not feasible for the timeline allowed for response to reviews.

In addition, the authors suggest that ApoE may be degraded by multiple autophagic pathways, including chaperone-mediated autophagy (CMA). But if ApoE is degraded by chaperone-mediated

© 2022. Published by The Company of Biologists under the terms of the Creative Commons Attribution License (https://creativecommons.org/licenses/by/4.0/).

autophagy post-Golgi, then there is currently a significant gap in the mechanism: how does ApoE escape from the secretory pathway into the cytoplasm, since CMA works on cytoplasmic proteins?

Although it is surprising that the CMA protein LAMP2A is required for the autophagic degradation of a secretory system protein such as APOE, the accumulation of APOE in neuronal LAMP2A knockdown mice has recently reported in vivo, further suggesting that LAMP2A plays a role in the autophagy of APOE. Of note, in the same study ATG7 knockdown was also found to significantly increase APOE levels, supporting our findings that multiple autophagy pathways may play a role. The following text has been added to the introduction and discussion lines highlighting this recent literature:

Lines 97-105:
"A recent study showed that CMA is inhibited in AD mouse models and down-regulated in human AD brain tissue. In CMA-inhibited AD mouse models, APOE accumulated significantly in brain tissue. Neuronal knockout of either LAMP2A or ATG7 in wild-type mouse brain was sufficient to increase levels of APOE in the insoluble proteome, suggesting that multiple autophagic pathways may play a role in the degradation of APOE (Bourdenx et al., 2021). We sought to build on these in vivo observations by investigating in vitro whether intracellular lysosomal degradation of APOE requires autophagy proteins involved in autophagosome formation, autophagosome fusion, or autophagosome-independent autophagy."

Lines 560-565:
"A recent study showed that LAMP2A-dependent CMA is inhibited in AD mouse models and down-regulated in human AD brain tissue. In LAMP2A-KO AD mouse models, APOE accumulated significantly in brain tissue (Bourdenx et al., 2021). Several SNPs in the LAMP2 gene are associated with significantly increased AD risk in human male APOE4 carriers, further suggesting that LAMP2-dependent autophagy of APOE4 may contribute to pathology"

Lines 589-593:
"In a recent study, quantitative proteomics of insoluble proteins revealed that APOE accumulates in the cortex of mice with either neuronal LAMP2A or ATG7 knocked down, suggesting that both proteins play a role in autophagic APOE degradation (Bourdenx et al., 2021). Cell-type or brain-region specific differences in which pathways degrade APOE merit further investigation. "

We have also added discussion of the literature regarding APOE escape into the cytoplasm and other organelles including mitochondria and the nucleus in lines 572-579: "If the LAMP2A-dependent mechanism that degrades APOE is CMA, it would be surprising that a secreted protein such as APOE could escape the secretory system into the cytosol to be recognized by Hsc70. There is some literature showing that APOE can escape into the cytoplasm in neurons (Chang et al., 2005; Huang et al., 2001; Lovestone et al., 1996) and hepatocytes (Hamilton et al., 1990), although one report did not observe cytoplasmic escape of APOE (DeMattos et al., 1999). APOE has also been observed outside the secretory system in mitochondria (Nakamura et al., 2009), mitochondria-derived vesicles (Roberts et al., 2021), and the nucleus (Parcon et al., 2017; Theendakara et al., 2016)."

Similarly, the degradation of internalized ApoE via LC3-associated autophagy (LANDO) is poorly developed. The authors point out that their results showing reduced internalization of ApoE upon knockdown of Rubicon or ATG7 in Figure 2 could be due to reduced endocytosis caused by impaired receptor recycling over time. The main evidence presented to rule this out is visualization of GFP-LC3A and ApoE3-mCh in Figure 2F. However, these images are not convincing, and no quantification of co-localization is included.

We agree that the mechanism of reduced internalization of APOE with Rubicon or ATG7 knockdown most likely involves reduced receptor recycling. This receptor recycling is facilitated by ATG8 recruitment to endosomes, as shown by Heckmann et al. 2019. This is discussed in lines 439-440: "While inhibition of LANDO does not inhibit the internalization of Aß initially, endocytosis over time is reduced due to impaired receptor recycling (Heckmann et al., 2019; Lucin et al., 2013)."

In order to further clarify that the mechanism of reduced fluorescence involves reduced internalization rather than accelerated autophagy with Rubicon knockdown, we added the following text and associated experiments:

© 2022. Published by The Company of Biologists under the terms of the Creative Commons Attribution License (https://creativecommons.org/licenses/by/4.0/).

Line 444-458: “Treatment of HepG2 cells with lysosomal de-acidifiers Baf or ammonium chloride (NH4Cl) significantly reduces internalization of APOE3-mCh (Fig. 8E), suggesting that reduced recycling of receptors may underlie the reduced APOE3-mCh fluorescence observed. A possible alternative mechanism of reduced fluorescence is that autophagy is stimulated by Rubicon knockdown to degrade more APOE3-mCh. In order to rule this out, a double knockdown of both Rubicon and ATG7 was performed (Fig. S8A). If Rubicon reduces fluorescence by stimulating macroautophagy, ATG7 knockdown should reduce the effect of Rubicon knockdown. To the contrary, we observed the same reduction of APOE3-mCh fluorescence in Rubicon and Rubicon+ATG7 knockdown cells, suggesting that these two knockdowns reduce fluorescence through the same mechanism (Fig. S8B). In order to directly visualize recruitment of LC3 family proteins to APOE-containing endocytic compartments in a LANDO-like process, ST14A cells, chosen for their flat morphology ideal for imaging, were treated with conditioned media containing APOE3-mCh or APOE4-mCh. Colocalization of APOE with LC3A/B was observed, with 17% of LC3A/B spots colocalizing with APOE3-mCh and 22% colocalizing with APOE4-mCH spots (Fig. S8C).”

Figure 2F has been removed from the manuscript, instead we performed confocal microscopy of ST14A cells that had endocytosed APOE3-mCh, and were then fixed and stained for endogenous LC3A/B. Results are shown in Figure 8C, and discussed in lines 453-458:

“In order to directly visualize recruitment of LC3 family proteins to APOE-containing endocytic compartments in a LANDO-like process, ST14A cells, chosen for their flat morphology ideal for imaging, were treated with conditioned media containing APOE3-mCh or APOE4-mCh. Colocalization of APOE with LC3A/B was observed, with 17% of LC3A/B spots colocalizing with APOE3-mCh and 22% colocalizing with APOE4-mCH spots (Fig. S8C).”

Another major point is that the authors switch between 3 different cell lines: HepG2, ST14A, and HEK293. The ST14A immortalized neuron line is appropriate for studying internalization of ApoE. However, neurons do not typically express high levels of ApoE (indeed, the authors could not detect endogenous ApoE in ST14A cells), so this does not seem like an appropriate choice for studying post-Golgi trafficking of ApoE. For future experiments, I recommend continuing to use HepG2 cells to study post-Golgi trafficking of ApoE, and/or switching to a cell line that better represents the cells that predominantly express and secrete ApoE in the brain (i.e., astrocytes and microglia).

We thank the reviewers for this suggestion. For the new figures that were produced to address these reviews; Figure S1Bii, Figure S2D, and Figure 4C, HepG2 cells were used exclusively. In Fig. S8C ST14A as this figure was designed to study endocytosis of APOE rather than expression.

In summary, the current manuscript contains interesting data about the turnover of ApoE, which is likely to be of value to the community. Unfortunately, neither of the two main observations (post-Golgi autophagy of ApoE by LAMP2-dependent autophagy and internalization of ApoE via LANDO) are well developed. Therefore, my opinion is that this manuscript will require major experimental work and restructuring in order to appropriate for publication in JCS.

We thank Reviewer 1 for noting the value of this work, and hope that our experimental efforts and restructuring of the text are satisfactory to address these concerns.

Reviewer 2 Advance summary and potential significance to field

In this submission by Fote and colleagues, the authors examine the degradation of the gene product of APOE, the major lipoprotein in the CNS, for which the presence of the APOE4 allele is one of the best known risk factors for late onset Alzheimer’s disease. Beginning with the premise that autophagosomes and enlarged endosomes accumulate in AD brain tissue, and especially for APOE4 carriers, the team investigates if APOE might be degraded by the lysosomal pathway autophagy, or through other LC3- associated processes. The authors use several immortalized cell lines, and brain tissue to identify which autophagy-related genes might be required to allow for APOE turnover. They find that a series of transcripts associated with autophagic and LC3-associated processes might affect APOE turnover, and several findings coalesce across multiple cell types, which is a strength.

We thank Reviewer 2 for noting the consistency of our findings regarding the accumulation of APOE with Baf or BFA treatment, or with LAMP2A knockdown across several cell lines.

© 2022. Published by The Company of Biologists under the terms of the Creative Commons Attribution License (https://creativecommons.org/licenses/by/4.0/).

However, since the continuity of experiments across cell types is limited, it is difficult to determine the overall conclusion of which if any autophagy pathway might be playing a role. Moreover, there are experiments that could be interpreted as contradictory, making it uncertain how to interpret the different findings across the different cell types, since it is unclear if these events are unique to certain cell types or not. This study addresses an understudied area, and the data suggest that there might be an isoform specific impact on/relationship with lysosomal degradation. Nonetheless in its current form, while there is a lot of different kinds of data presented, the study also doesn't quite mechanistically establish what underlies this difference, which may in part be improved by providing greater cohesion between the multiple data sets.

We thank Reviewer 2 for these suggestions. We have attempted to improve the cohesion of the manuscript by addressing the specific comments below.

Reviewer 2 Comments for the author
Although the comments below can help, a restructuring of the paper might also be helpful.

As noted above in response to Reviewer 1: in order to improve the continuity of the story, the figures and text have been rearranged to first show the differences in the autophagic degradation of the disease-associated isoform APOE4 (previously figures 5-7) followed by more mechanistic details regarding proteins involved in the autophagy of APOE (previously figures 1, 3, and 4) and finally figures having to do with the autophagy-protein dependent internalization of APOE (figures 2 and 8).

Overall comments
1. The presentation of the imaging data can be vastly improved, with grayscale images replacing the colored images.

We thank Reviewer 2 for this excellent suggestion and have converted images where possible to grayscale (merged multi-channel images to demonstrate co-localization were kept in color.)

2. Some of the statements were not quite accurate, and have been addressed in the comments below.
Figure 1 and related supplementary data:
1. The 'positive' data is placed in the figures, whereas the 'negative' data is placed in supplemental. To give a better picture of the data, it would be useful to show these data together. For example, LAMP2A and 2B should be shown together in the figures.

We thank the reviewer for this suggestion. We have moved Supplementary Figure 1D showing no change in APOE mRNA with LAMP2A knockdown to the main figure (Fig. 4G). Unfortunately we have been unable to investigate the effect of LAMP2B knockdown on APOE levels. We have purchased two sets of LAMP2B siRNA, including one found in the literature and another custom designed, and neither of these produced LAMP2B knockdown when validation was attempted by qPCR. Our LAMP2A siRNA produces LAMP2A knockdown without LAMP2B knockdown, shown in Figure 4G (previously Figure 1).

2. What is the outcome of Stx17 KD alone?

STX17 knockdown significantly increases APOE levels in HepG2 cells, see Figure 4E (previously Figure 1).

3. Please use endogenous LC3 immunofluorescence for co-localization

Colocalization of endogenous APOE and endogenous LC3A/B was assessed in HepG2 cells with Bafilomycin A1 or Brefeldin A treatment. Results are in Figure S 1Bii and Figure S2D. The following text was added:

Line 241-242:
"This dispersed pattern was also observed in HepG2 cells endogenously expressing APOE that were treated with BFA (Fig S1Bii)."
Line278-280:

© 2022. Published by The Company of Biologists under the terms of the Creative Commons Attribution License (https://creativecommons.org/licenses/by/4.0/).

"In HepG2 cells, 25% of LC3A/B positive vesicles colocalized with endogenously expressed APOE. Baf treatment significantly increased colocalization and number of APOE spots per cell (Fig. S 2D)."

4. Western blots examining protein levels (whenever possible) are important to ensure that functional changes are likely

Western blots showing siRNA knockdown of LAMP2A, STX17, and ATG7 in HepG2 cells are shown in Figure 4D-F (previously Figure 1 D-F). This figure also validates the use of siATG7 in Figure 8, previously Figure 2. Western blots showing shRNA knockdown of LAMP2A in ST14A cells is shown in Figure 6 (previously Figure 4) and shRNA knockdown of ATG7 in ST14A cells is shown in Supp Figure 4. Western blot validating knockdown of Rubicon alone or co-knockdown of Rubicon and ATG7 have been added to Fig S8A, with the following associated text in lines 437-438: "Knockdown of ATG7 and Rubicon were verified by qPCR (Fig 8C-D) and western blot (Fig 4F, Fig. S8A)."

5. Related text: line 170, given the hypothesis proposed for Figure 2, LC3-positive vesicle might be more appropriate.

Line 284-286 has been revised: "3-dimensional visualization of Z-stack images reveals that APOE3-mCh is completely engulfed in some LC3-positive vesicles (Supp Video 1)."

Figure 2 and related supplementary data:
1. The loss of Rubicon may also augment macroautophagy, hypothetically thereby driving degradation to occur faster (given the conclusion drawn at the end of Figure 1- see comment 5 above). Lysosome-inhibition to prevent degradation could be helpful here.

We thank Reviewer 2 for this helpful suggestion. We attempted to inhibit lysosomes using chloroquine concurrent with Rubicon knockdown to determine whether upregulated macroautophagy is responsible for the reduced fluorescence observed in siRubicon cells. However, red fluorescence is not adequately observed in either siCtrl or siRubicon cells with Chl treatment or with other lysosomal inhibitors (see Fig 8E). Instead, we performed double knockdown of ATG7 and Rubicon in the same cells. If Rubicon reduces fluorescence by stimulating autophagic degradation and ATG7 is required for macroautophagy (as well as LANDO), then concurrent knockdown should mitigate the reduction of fluorescence seen in siRubicon cells. To the contrary, we saw the same reduction in siRubicon and siRubicon+siATG7 cells, suggesting that these two knockdowns operate through the same mechanism.

The results are discussed in line 447-453: "A possible alternative mechanism of reduced fluorescence is that autophagy is stimulated by Rubicon knockdown to degrade more APOE3-mCh. In order to rule this out, a double knockdown of both Rubicon and ATG7 was performed (Fig. S8A). If Rubicon reduces fluorescence by stimulating macroautophagy, ATG7 knockdown should reduce the effect of Rubicon knockdown. To the contrary, we observed the same reduction of APOE3-mCh fluorescence in Rubicon and Rubicon+ATG7 knockdown cells, suggesting that these two knockdowns reduce fluorescence through the same mechanism (Fig. S8B)."

2. See fig 1 comment 4.

Western blots showing siRNA knockdown of LAMP2A, STX17, and ATG7 in HepG2 cells are shown in Figure 4D-F (previously Figure 1 D-F). This figure also validates the use of siATG7 in Figure 8, previously Figure 2. Validation of Rubicon knockdown has been added to Fig S8A.

3. See Fig 1 comment 3.
4. What is the role of other atg8 orthologs in the process? Given the previously described role of GABARAPs, this might be relevant here.

We performed co-localization analysis of endogenous LC3 and GABARAPL1 with endocytosed APOE using HepG2 cells. The results of our analysis of LC3 are included in Fig S8C. We found less colocalization with GABARPL1 (15%) and the images appeared unconvincing, therefore this was not included in the final supplementary figure.

5. Quantification is lacking for image based analyses (ex. Fig 2F).

© 2022. Published by The Company of Biologists under the terms of the Creative Commons Attribution License (https://creativecommons.org/licenses/by/4.0/).

Figure 3 and related supplementary data

We thank Reviewer 2 for this comment, Fig 2F has been removed from the manuscript. Figure 5 (previously Figure 3) has quantification of red and green fluorescence alongside the images in panels B, C, and E.

1. See comment Fig 2 #5. Images in 3B also look over saturated.

Incucyte live cell imaging system creates optimal microscopy settings automatically. Brightness of images in Figure 5 (previously Figure 3) were initially increased (equally throughout the figure) in order to demonstrate colors appropriately. The brightness has been reduced in representative images. The quantification of red and green fluorescence alongside the images in panels B, C, and E.

2. Associated text line 203: the dual tag does not necessarily indicate direct trafficking. Only that the tagged protein arrives to an acidified environment.

Line 294-295 (previously 203) was edited:
"To confirm that transfected APOE is trafficked into lysosomes, we generated a pH-sensitive dual-tagged human APOE3-mCh-SepHluorin (APOE-mCh-SepH) construct (Fig. 5A)."

Figure 4 and related supplementary data
1. Is there any correlation between level of KD and APOE3 levels (Fig 4A)?

There is a negative correlation between LAMP2A expression and APOE3 levels. A graph showing the correlation has been added to Figure 6A (previously Figure 4A) and text has been added to line 348-352: "APOE3-myc-flag protein levels were significantly increased by LAMP2A knockdown (Fig. 6A), and levels of APOE were negatively correlated with LAMP2A levels (Pearson's correlation coefficient -0.65, $p < 0.02$); fluorescence intensity of APOE3-mCh increased significantly (Fig. 6B)."

2. Lamp2B should also be revisited.

We regrettably are unable to find siRNA that effectively and specifically knocks down LAMP2B.

3. Although the addition of the LAMP2 KO mice is nice, given that it does not segregate between the different isoforms, it is difficult to make an isoform specific conclusion, which seems critical for the conclusion here.

Although we were unable to obtain LAMP2A knockout or knockdown mice for this study, a recently published paper shows that neuronal LAMP2A knockdown causes APOE accumulation in mouse brain tissue. As mentioned above, the results of this recent paper are now outlined in the discussion and introduction:

Lines 97-105:
"A recent study showed that CMA is inhibited in AD mouse models and down-regulated in human AD brain tissue. In CMA-inhibited AD mouse models, APOE accumulated significantly in brain tissue. Neuronal knockout of either LAMP2A or ATG7 was sufficient to increase levels of APOE in the insoluble proteome, suggesting that multiple autophagic pathways may play a role in the degradation of APOE (Bourdenx et al., 2021). We sought to build on these in vivo observations by investigating in vitro whether intracellular lysosomal degradation of APOE requires autophagy proteins involved in autophagosome formation, autophagosome fusion, or autophagosome-independent autophagy."

Lines 560-565:
"A recent study showed that LAMP2A-dependent CMA is inhibited in AD mouse models and down-regulated in human AD brain tissue. In LAMP2A-KO AD mouse models, APOE accumulated significantly in brain tissue (Bourdenx et al., 2021). Several SNPs in the LAMP2 gene are associated with significantly increased AD risk in human male APOE4 carriers, further suggesting that LAMP2-dependent autophagy of APOE4 may contribute to pathology"
Lines 589-593:

© 2022. Published by The Company of Biologists under the terms of the Creative Commons Attribution License (https://creativecommons.org/licenses/by/4.0/).

“In a recent study, quantitative proteomics of insoluble proteins revealed that APOE accumulates in the cortex of mice with either neuronal LAMP2A or ATG7 knocked down, suggesting that both proteins play a role in autophagic APOE degradation (Bourdenx et al., 2021). There may be cell-type or brain-region specific differences in which pathways are activated to degrade APOE, which merit further investigation."

Figure 5 and related supplementary data
1. It is difficult to ascertain where the work is going- the association of LAMP2A with CMA is strong, and this has not been completely ruled out by the previous experiments.

We agree our data does not rule out CMA as a contributing mechanism to APOE degradation, and that it is likely based on the increased levels of APOE with knockdown of LAMP2A, ATG7, or STX17, that multiple autophagic mechanisms are involved. This is supported by a recent study showing that neuronal LAMP2A or ATG7 knockdown in vivo increases APOE levels, as mentioned above.

As for macroautophagy, the lack of a rapamycin induced event is unexpected, since cargo that get degraded in an adaptor protein-dependent manner is also usually impacted by mTOR inhibition.

The following discussion of these results has been added to lines 594-601 in order to further discuss these unexpected results:
“We found that lysosomal degradation of APOE in HEK293 cells was not amplified by Rap on western analysis. Rap provides only partial inhibition of mTORc1(Nyfeler et al., 2011), and mTORc2, which may also contribute to autophagic regulation, is only sensitive to Rap with prolonged (< 24hr) treatment (Sarbassov et al., 2006). APOE may be targeted by a selective type of autophagy that is not activated by Rap; Rap has no effect on CMA (Finn et al., 2005) so it may not impact LAMP2A-mediated APOE degradation. APOE4 may induce the same macroautophagic pathway as Rap, preventing stimulation of further macroautophagic degradation with Rap treatment.”

It is also unclear, what exactly is the cargo being captured, if macroautophagy is the mechanism as implied by this section. Is it APOE or a vesicle containing APOE?

We have demonstrated co-localization of APOE with LC3-positive vesicles, as shown in Figure S2 D-E. Combined with the effect of ATG7 or STX17 knockdown this suggests that a macroautophagy mechanism underlies at least some APOE degradation. The only way we can think of to determine definitively whether APOE is contained within autophagosomes would be through electron microscopy that is not feasible for this study. However, as mentioned in response to reviewer 1 we have added discussion of the literature regarding APOE escape into the cytoplasm and other organelles including mitochondria and the nucleus in lines 572-579: “If the LAMP2A-dependent mechanism that degrades APOE is CMA, it would be surprising that a secreted protein such as APOE could escape the secretory system into the cytosol to be recognized by Hsc70. There is some literature showing that APOE can escape into the cytoplasm in neurons (Chang et al., 2005; Huang et al., 2001; Lovestone et al., 1996) and hepatocytes (Hamilton et al., 1990), although one report did not observe cytoplasmic escape of APOE (DeMattos et al., 1999). APOE has also been observed outside the secretory system in mitochondria (Nakamura et al., 2009), mitochondria-derived vesicles (Roberts et al., 2021), and the nucleus (Parcon et al., 2017; Theendakara et al., 2016).”

2. These experiments raise the need for understanding what type of vesicle is being examined when APOE and LC3 co-localize, and accumulate upon Baf treatment. Immunofluorescent staining for rab proteins might be helpful here.

Unfortunately all of the Rab5, Rab7, LC3 and GABARAPL1 antibodies that we have optimized for imaging are rabbit, thus we are unable to co-stain with more than one of them.

3. 3MA would impact all membrane trafficking events for which PI3KC3 would be present, suggesting that the concentration of 3MA might not have been sufficient (for which this compound is notorious). No control is run to determine this.

3MA has been removed from this figure and the associated text.

Figure 6: The rationale for suddenly adding APOE4 is lacking. Why was this not examined earlier?

© 2022. Published by The Company of Biologists under the terms of the Creative Commons Attribution License (https://creativecommons.org/licenses/by/4.0/).

As mentioned above, the figures and text have been rearranged to first show the differences in the autophagic degradation of the disease-associated isoform APOE4 (previously figures 5-7), in order to better emphasize how changes in autophagic degradation of APOE may be perturbed by the APOE4 isoform, which is associated with disease. This is followed by more mechanistic details regarding autophagy of APOE.

Figure 7 and related supplementary data:
1. Line 321: should be Fig. 7A and 7B

This line has been removed from the manuscript.

2. The presence of absence of Baf on LC3 levels can be assessed to see if further inhibition can be assessed.

We thank the reviewer for this suggestion, the impact of Baf levels on LC3 and APOE in these HEK cells can be found in Fig 2 (previously Fig 5). To make the results more clear, we have added to the explanation of these results in the text:

Line 145-148:
"Baf treatment significantly increased LC3 abundance in mCh-expressing cells, suggesting effective inhibition of the lysosome, but failed to significantly change LC3 abundance or ratio in APOE3-mCh or APOE4-mCh-expressing cells (Fig 1B), possibly suggesting that there is already a late-stage autophagic block at the level of the lysosome."

3. How these observation are influenced by the findings in Fig. 1, and the role of rbcn is unclear. The loss of Rbcn impeded packaging of APOE, and thus if packaging is central to its degradation, it is difficult to interpret these findings.

Although Figure 4 (previously figure 1) and Figure 1 (previously Figure 7) are in different cell lines (HEK 293 and HepG2), they reflect similar results; Baf and BFA increase APOE abundance in both cell lines. We are not sure how Rubicon would impede packaging of APOE; our hypothesis is that Rubicon knockdown impairs LANDO to inhibit internalization of APOE from the extracellular space.

Figure 8 and related supplementary data:
1. Although Rab7 was explored here, whether it would also be present for Figure 5 is unclear.

We thank the reviewer for this feedback. Since the HEK293 cells secrete, express, and endocytose fluorescent APOE, we suspect that there would indeed be some colocalization between APOE and Rab7 in HEK293 cells as well. However, in the HEK293 cells, it would be difficult to determine whether the fluorescent APOE colocalizing with Rab7 compartments originated from within the cells, through autophagy, vs. through endocytosis. With the conditioned media system used in Figure 8, in which media is collected from these HEK293 cells and added to ST14A or HepG2 cells, which are then imaged, we can examine the progress of APOE3 vs. APOE4 through the endosomal system and compare the localization of these isoforms to various endosomal compartments.

2. The quality of the imaging for 8C should be improved, especially for the conclusions drawn.

We have been unable to obtain a higher confocal objective to repeat this study at higher magnification. We have added "may be" our conclusions in line 402-404: "There was also a significantly higher intensity of Rab7 staining per cell in APOE4 treated cells (Fig. 7C), suggesting that late endosomes may be enlarged by internalization of APOE4" and removed "late endosomes" from line 463-464: "In this study, we found that APOE4 has increased lysosomal trafficking and accumulates in enlarged lysosomes, reflecting impaired clearance despite activated macroautophagy."

3. Proteomic analyses- the analyses were done through non quantitative approaches. Thus the similarity across different runs (Pearson's correlations) should be to determine variability of runs. It is unclear whether corrections for multiple comparisons have been made.
We report p values from ANOVA analysis comparing our replicates of the measured abundances in Supp Table 5; these values should supersede the need for a direct comparison of the variation

© 2022. Published by The Company of Biologists under the terms of the Creative Commons Attribution License (https://creativecommons.org/licenses/by/4.0/).

between replicates. Our proteomics analysis is consistent with the state of the art as described in Yaoyang et al. 2013 in Chemical Reviews. As is common in the field, we did not perform corrections for multiple comparisons.

Minor comments:
Line 176: PI3KC3 ‘usual function’ is not accurate. Rubicon can act to change in which complex PI3KC3 can reside.

We have changed line 432-434: “Rubicon can divert the Class III PI3K complex (PI3KC3) away from its function of initiating autophagosome formation, inhibiting macroautophagy and activating non-canonical autophagy instead (Wong et al., 2018).”

Second decision letter

MS ID#: JOCES/2021/258687

MS TITLE: Isoform-dependent lysosomal degradation and internalization of Apolipoprotein E requires autophagy proteins

AUTHORS: Gianna M. Fote, Nicolette R. Geller, Nikolaos Efstathiou, Nathan Hendricks, Demetrios G. Vavvas, Jack C. Reidling, Leslie M. Thompson, and Joan S. Steffan
ARTICLE TYPE: Research Article

I am happy to tell you that your manuscript has been accepted for publication in Journal of Cell Science, pending standard ethics checks. Please feel free to incorporate the minor comments made by reviewer 2 in your final version. It is not necessary, however.

Reviewer 1

Advance summary and potential significance to field

APOE4 is the strongest genetic risk factor for late-onset Alzheimer’s disease, yet little is known regarding how ApoE protein is intracellularly degraded. Previous work has demonstrated that ApoE is degraded in the lysosome, and that lysosomal accumulation of ApoE4 causes swollen lysosomes and defects in proteostasis.
Here, Fote et al demonstrate that ApoE can be degraded by autophagy after leaving the Golgi. Specifically the role of LAMP2A-mediated autophagy is demonstrated using multiple cell lines, which is a novel observation in the field. ApoE4 is shown to cause impaired autophagic flux and enlarged lysosomes. E4 was also found to have a slower rate of uptake through endocytosis than E3.

The experiments presented in this paper were well performed and presented, and quantification and statistical analyses are rigorous and transparent. Key experiments are replicated in multiple cell lines using different strategies for ApoE expression, strengthening support for the paper’s findings. The observation that ApoE is degraded by LAMP2A-mediated autophagy post-Golgi is a novel result in the field and important to our understanding of ApoE protein function.

Comments for the author

The authors have done a very substantial amount of work to improve this manuscript. They have addressed my main concerns by:
1) Restructuring the paper dramatically to improve the narrative flow.
2) Adding experimental evidence to show that they are in fact monitoring post-Golgi trafficking of ApoE rather than secretion followed by endocytosis.
3) Adding discussion to clarify their model for how ApoE could be degraded by CMA, i.e., after escape into the cytoplasm.
I recommend acceptance for publication in JCS.

© 2022. Published by The Company of Biologists under the terms of the Creative Commons Attribution License (https://creativecommons.org/licenses/by/4.0/).

<u>Reviewer 2</u>

Advance summary and potential significance to field

In this resubmission by the group of Joan Steffan, the authors examine the degradation pathways responsible for the turnover of ApoE isoforms. ApoE isoforms can play a significant role in the aging brain, indicated by the increased risk for the onset of AD associated with the isoform ApoE4. Gaining insight into the turnoer of the ApoE isoforms may allow us to gain insight into how these isoforms might differentially impact CNS health.

Comments for the author

In this resubmission by Fote and colleagues, the authors were highly responsive.
Most notable is the extensive rewriting of the manuscript, coupled by a greater focus on the mechanism of LAMP2A and its role in ApoE turnover. Given the recent work from the Cuervo lab, providing parallel evidence of the importance of this LAMP2 isoform, the work is timely and appropriate. Moreover, the authors also thoroughly addressed the previously noted concerns.
Minor comments Given the evolution of the paper, the title seems vague and doesn't represent the paper to the fullest. This reviewer suggests that LAMP2A be mentioned in the title rather than broadly indicate autophagy proteins.
At some point, bafilomycin is referred to as a lysosome de-acidifier. While not inaccurate, perhaps it can be referred to as an inhibitor of the V-ATPase, since it's administration would impact the acidity of all vesicles that express this pump.

© 2022. Published by The Company of Biologists under the terms of the Creative Commons Attribution License (https://creativecommons.org/licenses/by/4.0/).
